# Avidity‐Based Capture of PD‐L1‐Expressing Exosomes via Dendrimer–Peptide Conjugates: A Nanoengineered Platform for Enhanced Prediction of Immunotherapy Response

**DOI:** 10.1002/advs.202509270

**Published:** 2025-08-21

**Authors:** Jiah Lee, Dongjun Shin, Chae Yeon Son, Hanbit Kang, Jung Hyun Choi, Hyun Sung Park, Seha Bang, Lucia Kim, Tae Hee Lee, Hyuk Soo Eun, Michael J Poellmann, Woo‐jin Jeong, Dong Hyung Kim, Jun Hyeok Lim, Seungpyo Hong, Jiyoon Bu

**Affiliations:** ^1^ Department of Biological Sciences and Bioengineering Inha University Incheon 22212 Republic of Korea; ^2^ Division of Biomedical Metrology Korea Research Institute of Standards and Science 267 Gajeongno, Yuseong‐Gu Daejeon 34113 Republic of Korea; ^3^ Department of Pathology Inha University Hospital Inha University College of Medicine Incheon 22332 Republic of Korea; ^4^ Department of Biomedical Laboratory Science Daegu Health College Chang‐ui building, 15 Yeongsong‐ro, Buk‐gu Daegu 41453 Republic of Korea; ^5^ Department of Internal Medicine College of Medicine Chungnam National University Daejeon 35015 Republic of Korea; ^6^ Division of Pulmonology Department of Internal Medicine Inha University Hospital Inha University College of Medicine Incheon 22332 Republic of Korea; ^7^ Pharmaceutical Sciences Division School of Pharmacy University of Wisconsin‐Madison 777 Highland Ave. Madison WI 53705 USA; ^8^ Capio Biosciences Korea Hongcheon Gangwon‐do 25114 Republic of Korea; ^9^ Biohybrid Systems Research Center Inha University 100 Inha‐ro, Michuhol‐gu Incheon 22212 Republic of Korea

**Keywords:** avidity‐based capture, dendrimer‐peptide conjugates, exosomes, immunotherapy, multivalent binding

## Abstract

A major challenge in immunotherapy is the inability to reliably predict patient responses due to the lack of robust biomarkers. Programmed cell death‐ligand 1 (PD‐L1)–expressing exosomes represent a promising biomarker candidate; however, existing detection platforms lack the sensitivity and specificity required for clinical translation. It is hypothesized that an avidity‐based capture strategy utilizing dendrimer‐mediated multivalent binding will effectively enhance molecular avidity and improve the selective capture of PD‐L1‐expressing exosomes. Supporting this hypothesis, atomic force microscopy (AFM) revealed that dendrimer–peptide conjugates synthesized using generation 7 poly(amidoamine) dendrimers (G7‐pPDL1) exhibited ≈2.48‐fold higher binding avidity than conventional anti‐PD‐L1 antibodies (aPD‐L1), attributed to multivalent interactions. This increased avidity led to enhanced in vitro specificity and enabled 1.55‐fold greater sensitivity in capturing PD‐L1‐expressing exosomes, compared to aPD‐L1. Clinical validation using serum samples from patients undergoing immune checkpoint inhibitor therapy demonstrated that PD‐L1‐expressing exosomes captured using the G7‐pPD‐L1 surface more accurately predicted treatment response and outperformed tissue‐based PD‐L1 scoring in prognostic value. Additionally, this platform is compatible with existing biosensing technologies and enables real‐time exosome detection with a limit of detection as low as 9.6 × 10^1^ vesicles mL^−1^. Taken together, these findings highlight the versatility and clinical promise of this avidity‐based capture strategy for advancing precision immunotherapy.

## Graphical abstract

This study introduces a dendrimer‐peptide conjugate platform (G7‐pPDL1) for avidity‐based capture of PD‐L1‐expressing exosomes. The system exhibits enhanced binding kinetics and exosome capture efficiency, enabling accurate prediction of immunotherapy outcomes. Its integration with biosensors further demonstrates potential for sensitive, real‐time exosome detection and broad applicability in precision cancer immunotherapy.

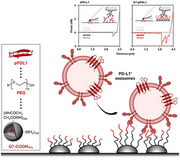

## 1 Introduction

Immune checkpoint inhibitors (ICIs) orchestrate the body's innate immune system by blocking inhibitory signals between immune cells and cancer cells.^[^ ^1^ ^]^ Since the approval of the first ICI, ipilimumab, the U.S. Food and Drug Administration (FDA) has approved ten ICIs, each showing therapeutic benefit across multiple malignancies.^[^ ^2^ ^]^ Among various immune checkpoint molecules, programmed cell death protein‐1 (PD‐1) and its counter ligand, programmed cell death‐ligand 1 (PD‐L1), are the most well‐established targets for immune checkpoint inhibition. Cancer cells exploit the PD‐1/PD‐L1 pathway by overexpressing PD‐L1 to evade T‐cell recognition and suppress antitumor immunity.^[^ ^3^ ^]^ Emerging evidence suggests that disrupting this signaling pathway through PD‐1 or PD‐L1 blockade substantially improves clinical outcomes across various cancers.^[^ ^4^ ^]^

However, overall response rates to ICIs remain limited and largely confined to specific patient subsets. For example, treatment with anti‐PD‐(L)1 monotherapy yields overall response rates of only 8–13% in tumors with low PD‐L1 expression.^[^ ^5^, ^6^ ^]^ ICIs may also induce immune‐related adverse events (irAEs), particularly in non‐responders.^[^ ^7^ ^]^ Due to such variable responses dependent on patients’ immunological and clinical profiles, numerous predictive biomarkers have been explored to enhance the therapeutic outcomes of ICIs.

Currently, PD‐L1 expression in tumor biopsies remains the clinical standard for predicting response to PD‐1/PD‐L1 blockade. However, PD‐L1 expression is highly heterogeneous both within and between tumors. Intra‐ and inter‐tumoral heterogeneity of PD‐L1 expression has been observed in 78% and 53% of non‐small cell lung cancer (NSCLC) patients, respectively.^[^ ^8^ ^]^ This variability contributes to inconsistent correlations between PD‐L1 expression levels and clinical outcomes following ICI treatment.^[^ ^8^ ^]^ Moreover, tissue‐based analysis requires sufficiently high‐quality samples, which are often unavailable in advanced‐stage disease—highlighting the need for more reliable, accessible biomarkers.

Exosomes have emerged as promising tumor biomarkers due to their overall abundance in bodily fluids and their role in intercellular communication.^[^ ^9^ ^]^ Secreted by nearly all cell types, exosomes carry molecular cargo that reflects their cellular origin.^[^ ^10^, ^11^, ^12^ ^]^ Tumor‐derived exosomes offer a non‐invasive means to access real‐time information from the tumor microenvironment, capturing spatial and temporal heterogeneity. Among their molecular contents, PD‐L1 on exosomes is found at substantially higher levels than its soluble form, making exosomal PD‐L1 a particularly informative biomarker.^[^ ^13^, ^14^ ^]^ Clinical studies have shown that baseline exosomal PD‐L1 better stratifies responders and non‐responders to ICIs than soluble PD‐L1, with higher pre‐treatment levels associated with poorer outcomes.^[^ ^15^, ^16^ ^]^

Despite these advantages, the utility of exosomal PD‐L1 is limited by the low abundance of tumor‐derived exosomes relative to the total vesicle population in circulation. The vast majority of exosomes originate from non‐malignant cells, making the selective detection technically challenging. Conventional antibody‐based detection platforms—including ELISA and western blotting—lack the sensitivity and specificity needed to distinguish tumor‐derived vesicles from the background. Moreover, the weak and transient nature of ligand‐receptor interactions in traditional assays comprises capture efficiency and diagnostic accuracy.

**Figure 1:**
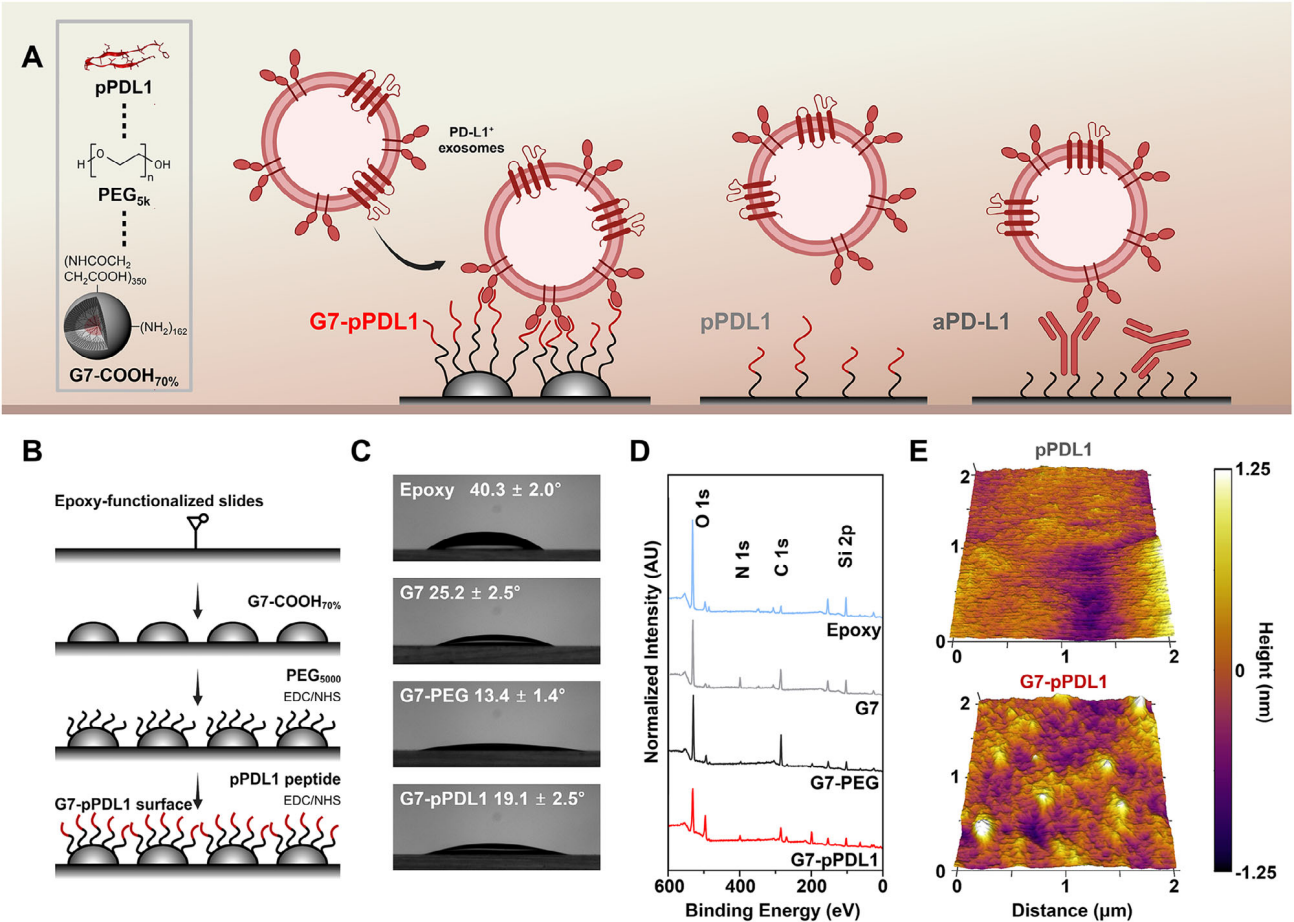
Overview of the avidity‐based capture surface (G7‐pPDL1) for the detection of PD‐L1^+^ exosomes: A) Schematic illustration of the G7‐pPDL1 surface designed to enhance PD‐L1^+^ exosome capture via multivalent binding interactions. B) Stepwise surface modification process: sequential immobilization of G7 PAMAM dendrimers, PEG_5000_, and PD‐L1–binding peptides on epoxide‐functionalized glass slides. C) Contact angle measurements showing changes in surface wettability after each modification step. D) X‐ray photoelectron spectroscopy (XPS) confirms changes in atomic composition corresponding to each layer. E) AFM‐based surface topography analysis comparing pPDL1 and G7‐pPDL1 surfaces, confirming successful dendrimer immobilization via increased surface roughness.

To address these limitations, we devised a high‐affinity nanostructured platform for capturing PD‐L1‐expressing exosomes by leveraging multivalent binding interactions (**Figure** **1**A). While peptides are widely used in biomedical applications due to their target specificity and synthetic accessibility, they often exhibit low targeting efficiency toward their molecular targets.^[^ ^17^ ^]^ Here, we hypothesized that incorporating a hierarchical multivalent architecture with peptides would markedly enhance in vitro binding affinity and maximize the capture of exosomes expressing the target protein.^[^ ^18^ ^]^ To test this hypothesis, we engineered a PD‐L1^+^ exosome capture surface composed of generation 7 (G7) poly(amidoamine) PAMAM dendrimers conjugated with our previously developed PD‐L1‐binding peptides (G7‐pPDL1).^[^ ^19^, ^20^ ^]^ Using atomic force microscopy (AFM)‐based nanomechanical analysis, we confirmed that dendrimer‐mediated multivalent binding significantly enhanced the interaction strength with a recombinant PD‐L1‐coated AFM probe, as evidenced by multiple discrete binding events occurring during the measurement. We further validated, through multiple assays, that this enhanced molecular interaction translated into improved sensitivity and specificity for isolating PD‐L1‐expressing exosomes. Using patient‐derived plasma samples, we demonstrated the clinical utility of our platform by comparing the predictive performance of PD‐L1^+^ exosomes with that of conventional tissue PD‐L1 immunohistochemical (IHC) staining. Additionally, we demonstrated the versatility of the avidity‐based capture strategy by integrating it into existing biosensing platforms, where it outperformed antibody‐based systems in sensitivity. Taken together, these findings highlight the potential of an avidity‐based nanoplatform to enable clinically meaningful exosome analysis for monitoring immunotherapy and predicting therapeutic response.

## 2 Results

### 2.1 Engineering of Avidity‐Based PD‐L1^+^ Exosome Capture Surface

The avidity‐based PD‐L1^+^ exosome capture surface (G7‐pPDL1) consists of multiple polymer layers engineered to minimize nonspecific binding, enable multivalency, and provide structural flexibility for peptide presentation (Figure 1A). Briefly, the surface was prepared by sequential immobilization of 70% carboxylated G7 PAMAM dendrimers, 5 kDa poly(ethylene glycol) (PEG), and pPDL1 peptides onto an epoxide‐coated microscope slide (Figure 1B). Note that partial carboxylation of dendrimers was confirmed using ^1^H NMR (Figure S1, Supporting Information), while the molecular weight and purity of pPDL1 were validated using matrix‐assisted laser desorption/ionization time‐of‐flight (MALDI‐TOF) mass spectrometry and high‐performance liquid chromatography (HPLC) (Figures S2 and S3, Supporting Information).

The immobilization of each polymer layer upon surface modification was verified by changes in surface wettability via sessile drop contact angle measurements (Figure 1C; Figure S4, Supporting Information) and by surface atomic composition analysis using X‐ray photoelectron spectroscopy (XPS) (Figure 1D; Figure S5, Supporting Information). Additionally, a significant increase in surface roughness following dendrimer presentation further supported successful surface modification (Figure 1E). To evaluate the enhanced binding kinetics and exosome capture performance of the G7‐pPDL1 surface, PEGylated surfaces functionalized with either pPDL1 or aPD‐L1 were prepared in parallel, while a scrambled peptide‐conjugated dendrimer surface (G7‐pPDL1_scr_) served as a negative control.

### 2.2 Nanomechanical Analysis of G7‐pPDL1 Reveals Enhanced Binding Kinetics

AFM force spectroscopy was employed to investigate the nanoscale mechanical interactions between the G7‐pPDL1 surface and PD‐L1^+^ exosomes. Recombinant PD‐L1 was immobilized on an AFM probe with a nominal diameter of ≈100 nm, approximating the size of a single exosome. Unlike our previous study, in which peptides and antibodies were compared at equal mass,^[^ ^20^ ^]^ the capture surfaces in this study were functionalized with equimolar amounts of aPD‐L1 and pPDL1. This design enabled to evaluate whether peptides—despite their lower monovalent affinity—could achieve enhanced binding comparable to antibodies via dendrimer‐mediated multivalent interactions.

**Figure 2:**
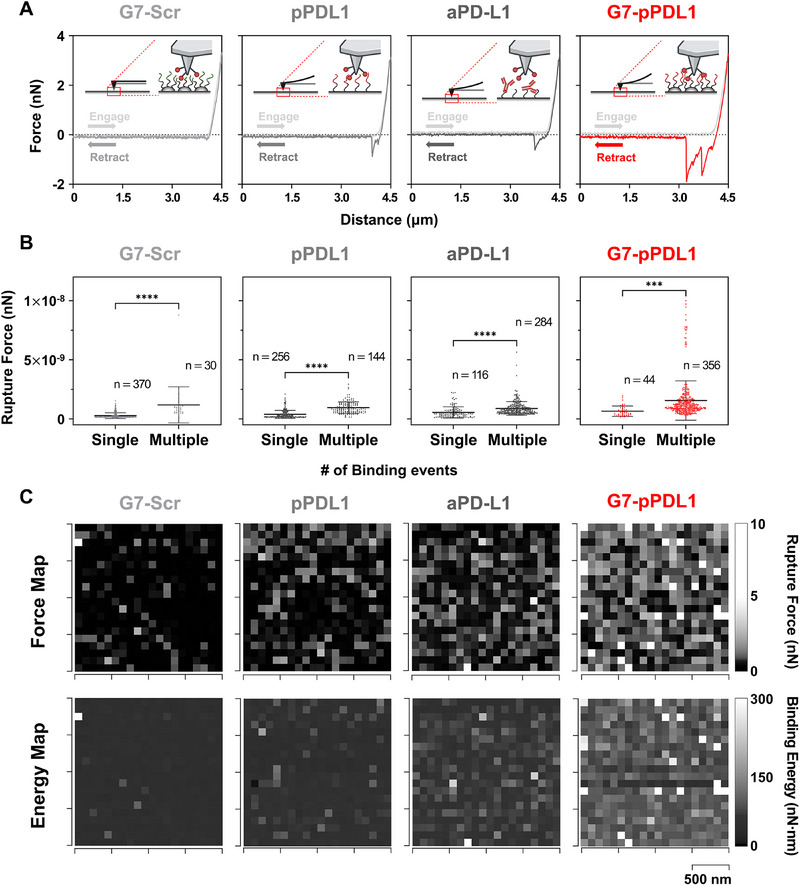
Characterization of binding kinetics of the G7‐pPDL1 surface using AFM force spectroscopy. A) Representative FD curves (n = 400) obtained from G7‐pPDL1_scr_, pPDL1‐, aPD‐L1–, and G7‐pPDL1–functionalized surfaces. The G7‐pPDL1 surface shows multiple discrete rupture events, indicative of multivalent binding. B) Quantification of distinct binding events per FD curve and comparison of rupture forces for single‐ versus multiple‐binding events across surfaces. Data represent mean ± SD; *n* indicates the number of curves analyzed per group. C) Force and energy heatmaps obtained from AFM adhesion force mapping over a 2 µm × 2 µm area, illustrating spatial variation in rupture force and adhesion energy. Note that the significance levels are indicated as ^**^ *p* < 0.001 and ^****^ *p* < 0.0001, determined by two‐sided Student's *t*‐tests or Mann–Whitney *U* tests, depending on whether the data met assumptions of normality.

A total of 20 × 20 force–distance (FD) curves were acquired over a 2 × 2 µm^2^ area on each surface. Representative FD curves are shown in **Figure** **2**A. The G7‐pPDL1 surface exhibited multivalent binding interactions more frequently than the comparison surfaces, as indicated by two or more abrupt force changes (threshold: 300 pN) during the retraction phase. A threshold of 300 pN was established based on the observation that such adhesion forces were rarely detected on the negative control surface (G7‐pPDL1_scr_). Specifically, multiple binding events were observed in 89.0% of FD curves obtained from the G7‐pPDL1 surface (Figure 2B). These events displayed significantly higher adhesion forces compared to single‐binding events (654.9 ± 432.6 pN vs 1558.3 ± 1662.8 pN; *p* < 0.001). While pPDL1‐ and aPD‐L1‐functionalized surfaces also showed elevated adhesion forces during multiple binding events, their occurrence was markedly lower (36.0% for pPDL1 and 71.0% for aPD‐L1). It should also be noted that the G7‐pPDL1_scr_ surface exhibited multiple binding events in only 7.5% of FD curves, indicating that the interactions observed on G7‐pPDL1 were specific rather than due to nonspecific binding.

As a result of multivalent interactions between pPDL1 and recombinant PD‐L1 immobilized on the AFM probe, the G7‐pPDL1 surface exhibited the highest mean maximum adhesion force among all tested surfaces (Figure 2C; Figure S6, Supporting Information). Specifically, the mean maximum adhesion force on G7‐pPDL1 was 1470.8 ± 1640.8 pN, significantly greater than that observed on aPD‐L1 (790.5 ± 570.6 pN; *p* < 0.0001) and pPDL1 (594.0 ± 478.0 pN; *p* < 0.0001) surfaces. The dendrimer‐mediated multivalent binding not only enhanced peak adhesion forces but also prolonged interactions with the target molecules. Notably, the differences in adhesion energy—defined as the cumulative product of force and separation distance—were also pronounced. The G7‐pPDL1 surface demonstrated an adhesion energy of 106.4 ± 76.6 pN·nm, significantly higher than that of aPD‐L1 (66.8 ± 27.3 pN·nm; *p* < 0.0001) and pPDL1 (51.5 ± 14.3 pN·nm; *p* < 0.0001) surfaces. In addition, the mean maximum adhesion force (350.9 ± 527.8 pN) measured on the negative control surface (G7‐pPDL1_scr_) was near the threshold (300 pN), indicating minimal nonspecific binding by the dendrimer itself. Collectively, these findings support our hypothesis that avidity‐based capture via dendrimer‐peptide conjugates substantially enhances both binding strength and interaction stability with target biomolecules, demonstrating their potential to improve the capture efficiency of PD‐L1‐expressing exosomes.

### 2.3 Multivalent Binding Interaction Enhances In Vitro Targeting

**Figure 3:**
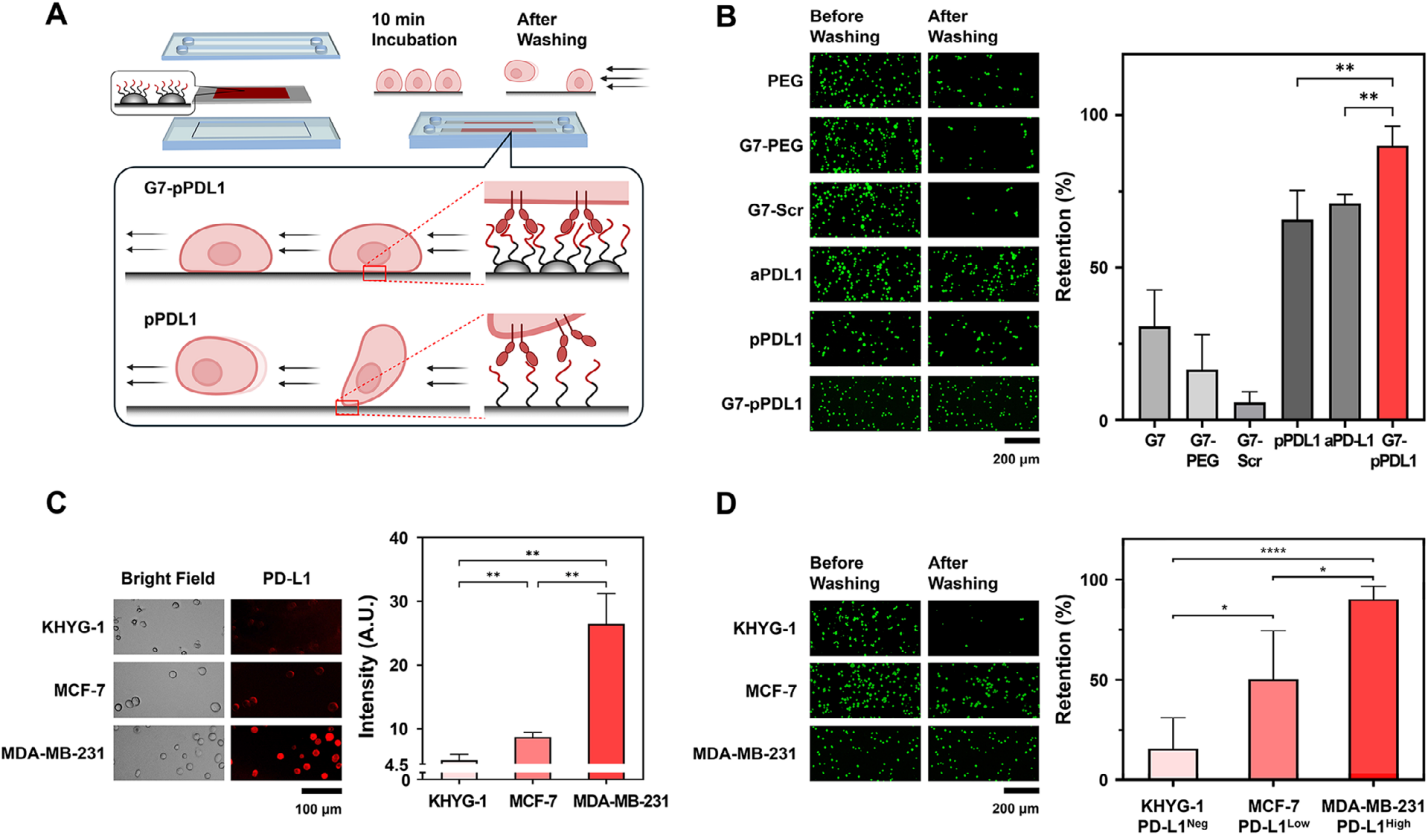
Translation of avidity‐enhanced binding kinetics to in vitro cell adhesion analyzed using a microfluidic‐based cell retention assay. A) Schematic representation of the cell retention assay for analyzing in vitro binding kinetics of G7‐pPDL1. B) Retention of PD‐L1^High^ MDA‐MB‐231 cells on surfaces functionalized with various PD‐L1 capture agents, including pPDL1, aPD‐L1, and G7‐pPDL1, as well as negative control groups (PEG, G7‐PEG, and G7‐pPDL1_scr_). C) Immunofluorescence analysis of PD‐L1 expression in PD‐L1^Negative^ KHYG‐1, PD‐L1^Low^ MCF‐7, and PD‐L1^High^ MDA‐MB‐231 cells. D) PD‐L1–dependent cell captures selectivity of G7‐pPDL1, analyzed by retention of different cell lines with varying PD‐L1 expression levels. Note that the significance levels are indicated as ^*^ *p* < 0.05, ^**^ *p* < 0.01, and ^***^ *p* < 0.001, determined by two‐sided Student's *t*‐test (*n* ≥ 3 for all experiments). Data are presented as mean ± SD from at least three independent experiments.

Prior to applying the G7‐pPDL1 surfaces for PD‐L1‐expressing exosome capture, a microfluidic‐based cell retention assay was performed to verify that the strong nanoscale binding avidity of G7‐pPDL1 could be translated into enhanced capture of intact biomolecular targets. As shown in **Figure** **3**A and Figure S7 (Supporting Information), a flow chamber equipped with capture surfaces functionalized with G7‐pPDL1, pPDL1, aPD‐L1, or various negative controls was used for the assay.^[^ ^21^ ^]^ Briefly, following 10 min of incubation within the flow chamber, retained cells were quantified after washing the chamber under a shear stress of 100 µL min^−1^ for 10 min. Detailed procedures are described in the Supporting Information.

As shown in Figure 3B, retention of PD‐L1^High^ MDA‐MB‐231 cells was significantly greater on G7‐pPDL1 surfaces (90.0 ± 5.5%) compared to pPDL1 (71.1 ± 2.3%; *p* = 0.0054) or aPD‐L1 (65.7 ± 7.8%; *p* = 0.0097) surfaces, indicating successful translation of avidity‐enhanced binding kinetics into in vitro cell adhesion. In contrast, surfaces lacking specific capture agents—including PEG, G7‐PEG, and G7‐pPDL1_scr_—showed consistently low cell retention, all below 30.7%. Among these, G7‐pPDL1_scr_ exhibited the lowest retention (5.7 ± 3.0%), suggesting that the multilayer polymer architecture not only enhances overall binding capacity but also effectively suppresses nonspecific adsorption.

To further assess the PD‐L1‐dependent binding selectivity of G7‐pPDL1, we compared the retention of three cell lines with varying PD‐L1 expression levels: MDA‐MB‐231 (PD‐L1^High^), MCF‐7 (PD‐L1^Low^), and KHYG‐1 (PD‐L1^Negative^) (Figure 3C). As illustrated in Figure 3D, the cell retention rate strongly correlated with PD‐L1 expression. Notably, the PD‐L1^Negative^ KHYG‐1 cells exhibited minimal retention (15.7 ± 14.1%), comparable to that observed on surfaces lacking PD‐L1 capture agents, thereby confirming the high specificity of G7‐pPDL1 for its intended target. Taken together, these findings demonstrate that the enhanced binding kinetics observed at the nanoscale effectively translate into selective in vitro cell capture, validating G7‐pPDL1 surfaces as a functional platform for targeting biomolecules.

### 2.4 Multivalent Binding Enhances the Capture of PD‐L1–Expressing Exosomes

G7‐pPDL1 surfaces were subsequently applied for the capture of PD‐L1‐expressing exosomes. Exosomes derived from PD‐L1^High^ MDA‐MB‐231 cells (≈5 x 10^10^ vesicles mL^−1^; ≈2 µL mm^−2^) were incubated on G7‐pPDL1, aPD‐L1, and pPDL1 surfaces for 3 h, and their capture efficiency was quantitatively compared. To evaluate exosome capture, three complementary assays were employed as previously described^[^ ^20^ ^]^: 1) membrane staining with the lipophilic green fluorescent dye DiO, 2) total protein quantification using the bicinchoninic acid (BCA) assay, and 3) nanoparticle tracking analysis (NTA).

**Figure 4:**
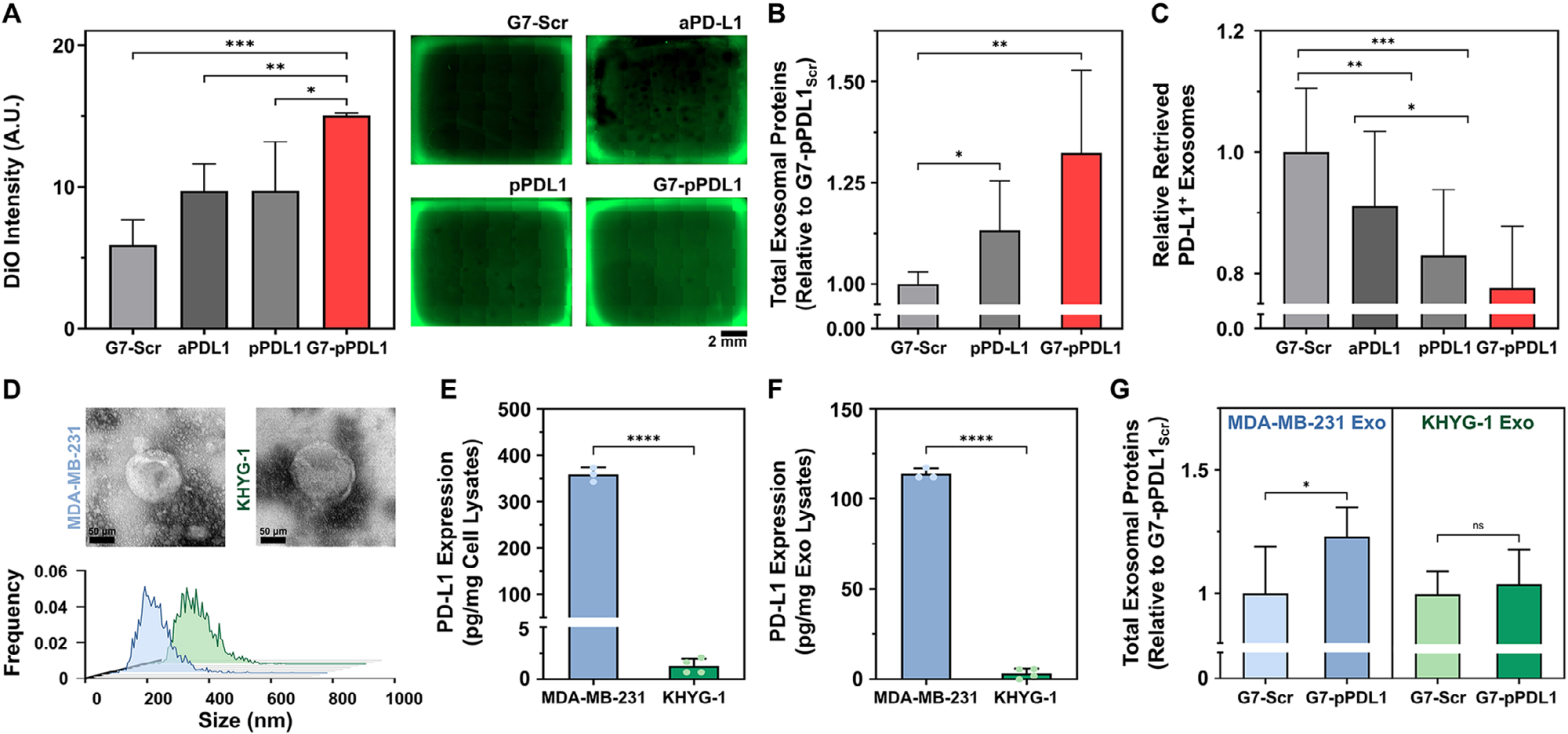
Multivalent binding interaction enhances the capture of PD‐L1‐expressing exosomes. The capture sensitivity and specificity of PD‐L1‐expressing exosomes on G7‐pPDL1 surfaces were compared with aPD‐L1, pPDL1, and G7‐pPDL1_scr_ surfaces. A) Quantification of captured exosomes derived from PD‐L1^High^ MDA‐MB‐231 cells using DiO membrane staining. Representative fluorescence images are shown on the right. B) BCA assay quantifying the total exosomal protein captured from MDA‐MB‐231‐derived exosomes on each surface. aPD‐L1 was excluded due to signal interference from antibody components. C) NTA analysis of MDA‐MB‐231–derived exosomes remaining in the post‐capture supernatant, assessing capture efficiency. D) TEM images and NTA size distribution profiles of exosomes derived from MDA‐MB‐231 and KHYG‐1 cells, showing comparable morphology and size distributions. E, F) ELISA quantification of PD‐L1 expression in parental cells and corresponding exosomes. G) BCA assay quantifying KHYG‐1‐derived exosomes captured on G7‐pPDL1 versus G7‐pPDL1_scr_ surfaces. Note that the significance levels are indicated as ^*^ *p* < 0.05, ^**^ *p* < 0.01, ^***^ *p* < 0.001, and ^****^ *p* < 0.0001, determined by two‐sided Student's *t*‐test (*n* ≥ 3 for all experiments). Data are presented as mean ± SD from at least three independent experiments.

As demonstrated in **Figure** **4**A, capture of PD‐L1^High^ exosomes was highest on the G7‐pPDL1 surface, as indicated by the strongest DiO fluorescence signal. Specifically, the fluorescence intensity of exosomes captured on G7‐pPDL1 surfaces was 15.0 ± 0.1 A.U., significantly exceeding that observed on aPD‐L1 (9.7 ± 1.6 A.U.; *p* = 0.0089) or pPDL1 (9.7 ± 3.0 A.U.; *p* = 0.0485) surfaces. A similar trend was observed in the BCA assay (Figure 4B), where the amount of exosomal protein captured on G7‐pPDL1 surfaces was ≈1.17‐fold greater than that on pPDL1 surfaces (*p* = 0.078). Note that due to signal interference from antibody components, aPD‐L1 was excluded from the BCA assay comparison. NTA analysis further supported these results, revealing a reduced number of vesicles remaining in the post‐capture supernatant (Figure 4C), indicative of higher exosome captures on the G7‐pPDL1 surface. Although statistical significance was modest, G7‐pPDL1 consistently demonstrated 1.17‐fold (*p* = 0.0221) and 1.07‐fold (*p* = 0.3175) higher capture of MDA‐MB‐231‐derived exosomes compared to aPD‐L1 and pPDL1 surfaces, respectively. Of note, exosome capture on the G7‐pPDL1_scr_ surface was negligible across all three assays, further confirming that the dendrimer structure alone does not contribute to PD‐L1^+^ exosome capture.

To further validate specificity, exosomes derived from PD‐L1^Negative^ KHYG‐1 cells were used as a negative control. Transmission electron microscopy (TEM), NTA, and dynamic light scattering (DLS) analyses demonstrated no significant differences in the physical characteristics of exosomes derived from MDA‐MB‐231 and KHYG‐1 cells (Figure 4D; Figure S8, Supporting Information). In contrast, ELISA revealed significantly lower surface PD‐L1 expression on KHYG‐1‐derived exosomes, consistent with expression profiles observed in the parental cell lines (Figure 4E,F). When KHYG‐1‐derived exosomes were used to assess the capture specificity of G7‐pPDL1, BCA analysis showed negligible differences in the amount of exosomal protein captured on G7‐pPDL1 versus G7‐pPDL1_scr_ surfaces (*p* = 0.6507), further confirming the high specificity of G7‐pPDL1 for PD‐L1^+^ exosome capture (Figure 4G). Moreover, only a markedly weak DiO fluorescence signal was detected when KHYG‐1‐derived exosomes labeled with DiO were spiked into human serum and incubated on the G7‐pPDL1 surface. Specifically, the fluorescence intensity obtained from the G7‐pPDL1 surface was not statistically different from that of the G7‐pPDL1_scr_ surface (0.92‐fold; *p* = 0.576) (Figure S9, Supporting Information). Altogether, these findings demonstrate that the dendrimer‐peptide conjugates enable selective and high‐efficiency capture of PD‐L1‐expressing exosomes, validating the successful translation of nanoscale binding avidity into functional exosome isolation.

### 2.5 Clinical Utility of PD‐L1^+^ Exosomes Captured via Avidity‐Based Platform

The clinical utility of PD‐L1^+^ exosomes captured using our avidity‐based platform (G7‐pPDL1 surface) was evaluated using serum samples obtained from 7 healthy donors, 30 hepatocellular carcinoma (HCC) patients, and 15 lung cancer patients (Tables S1 and S2, Supporting Information). Among the HCC cohort, patients underwent various treatment modalities, including radiofrequency ablation (RFA), trans arterial chemoembolization (TACE), surgical resection, or chemotherapy with sorafenib. In contrast, all lung cancer patients were treated with ICIs, specifically pembrolizumab or atezolizumab. Total serum exosomes were isolated using ExoQuick according to the manufacturer's instructions, followed by incubation on the G7‐pPDL1 capture surface for 3 h. Prior to clinical application, successful exosome isolation by ExoQuick was confirmed using TEM, NTA, and western blot analysis (Figure S10, Supporting Information). The PD‐L1^+^ exosome enrichment efficiency was then calculated by comparing the amount of total exosomal protein captured on G7‐pPDL1 surfaces to that captured on negative control surfaces (G7‐pPDL1_scr_).

**Figure 5:**
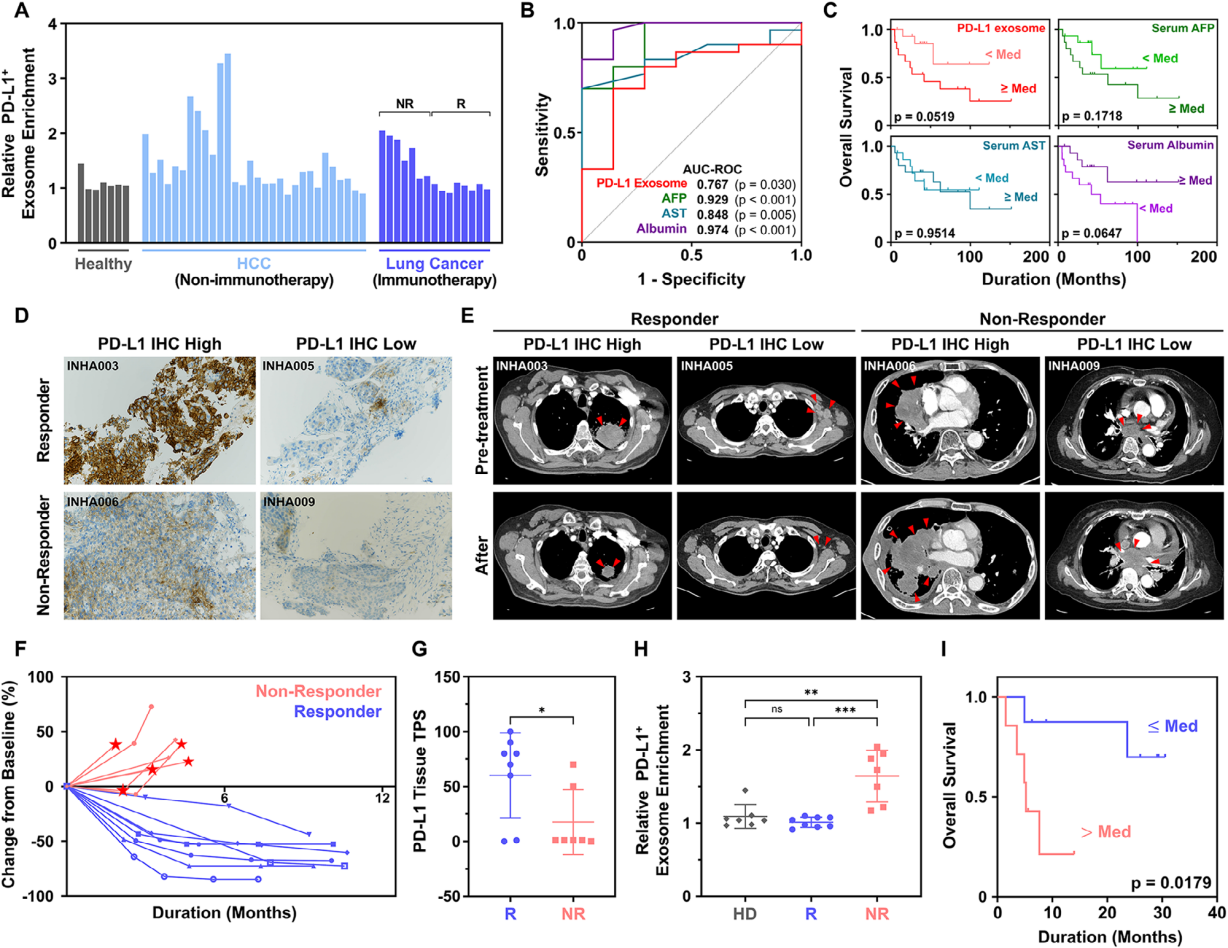
Clinical utility of PD‐L1^+^ exosomes captured via an avidity‐based platform. A) Relative PD‐L1⁺ exosome expression levels in pre‐treatment serum samples obtained from 7 healthy donors, 30 HCC patients, and 15 lung cancer patients subsequently treated with ICIs. B) ROC analysis comparing the diagnostic performance of PD‐L1⁺ exosomes and conventional serum biomarkers for HCC, including AFP, AST, and albumin. C) Kaplan–Meier analysis of OS in HCC patients stratified by the median levels of PD‐L1⁺ exosomes, AFP, AST, and albumin. D) Representative IHC images showing PD‐L1 expression in tumor tissues of responders and non‐responders to ICI therapy in lung cancer patients. E) CT scans of responders and non‐responders before and after ICI treatment, indicating tumor size changes. F) Waterfall plot showing percentage change in tumor burden from baseline during ICI treatment for each patient. Red asterisks indicate non‐responders with new metastasis found during therapy. G) Comparison of tumor PD‐L1 IHC scores between responders (R) and non‐responders (NR). H) Relative PD‐L1⁺ exosome levels in healthy donors (HD), responders (R), and non‐responders (NR), showing significant elevation in non‐responders. I) Kaplan–Meier survival analysis of lung cancer patients stratified by median PD‐L1⁺ exosome expression, showing significantly shorter OS in patients with high PD‐L1⁺ exosome levels. Note that the significance levels are indicated as *^ns^* not significant*, p* < 0.05, ^**^ *p* < 0.01, and ^***^ *p* < 0.001, determined by two‐sided Student's *t*‐test. Data represent mean ± SD.

As shown in **Figure** **5**A, the relative PD‐L1^+^ exosome enrichment efficiency differed across cohorts. In HCC patients, PD‐L1^+^ exosome enrichment efficiency was higher than that observed in healthy donors (1.09 ± 0.15 vs 1.50 ± 0.65; *p* = 0.1176). Consequently, the diagnostic capability of PD‐L1^+^ exosomes captured on G7‐pPDL1, as evaluated by ROC analysis (Figure 5B), demonstrated an AUC of 0.767 (*p* = 0.030), indicating moderate diagnostic performance. This was not as robust as conventional HCC‐associated serum protein markers, including α‐fetoprotein (AFP;AUC = 0.929; *p* < 0.001), aspartate aminotransferase (AST; AUC = 0.848; *p* = 0.005), and albumin (AUC = 0.974; *p* < 0.001). However, given that reported PD‐L1 tissue positivity in HCC ranges from ≈17–30%, these findings are consistent with clinical expectations.^[^ ^22^, ^23^ ^]^

In contrast, when evaluating the prognostic potential, PD‐L1^+^ exosomes outperformed conventional serum markers in predicting patient survival outcomes (Figure 5C). Using the median value of each biomarker as the threshold, patients with high PD‐L1^+^ exosomes exhibited a significantly shorter overall survival (OS; 65.52 ± 15.65 months) compared to those with low PD‐L1^+^ exosomes (93.88 ± 15.32 months; *p* = 0.052). Although patients with elevated serum AFP or reduced albumin levels also exhibited trends toward poorer survival (Table S3, Supporting Information), the associations were less statistically significant than those observed for PD‐L1^+^ exosomes.

The predictive capability of PD‐L1⁺ exosomes isolated using our system was evaluated for estimating therapeutic outcomes following ICI treatment and compared with the conventional tissue PD‐L1 tumor proportion score (TPS) (Figure 5D).^[^ ^24^ ^]^ Among the fifteen lung cancer patients included in this study, eight patients demonstrated tumor size reduction following ICI therapy (Figure 5E,F), corresponding to partial responses (PR; responders). The remaining seven patients exhibited progressive or stable disease (PD or SD; non‐responders), including five who developed new metastases during therapy (indicated by asterisks in Figure 5F), reflecting tumor progression despite treatment.

As expected, tissue PD‐L1 TPS was higher in responders than in non‐responders (60.1 ± 38.7% vs 17.7 ± 29.5%) with a significance level of *p* = 0.035 (Figure 5G). Notably, PD‐L1⁺ exosome analysis outperformed conventional TPS (Figure 5H), revealing significantly higher levels in non‐responders (1.64 ± 0.35) than in responders (1.01 ± 0.07), with stronger statistical discrimination (*p* = 0.003), consistent with previous reports.^[^ ^15^, ^16^ ^]^ Importantly, the lowest PD‐L1⁺ exosome level among non‐responders exceeded the highest level observed in responders, yielding an AUC‐ROC of 1.000 (*p* < 0.001). It should also be noted that PD‐L1⁺ exosome expression levels did not differ significantly between responders and healthy donors, whereas non‐responders consistently exhibited significantly elevated levels.

Patients with high PD‐L1⁺ exosome expression also demonstrated significantly poorer OS, as shown in Figure 5I. Specifically, patients with PD‐L1⁺ exosome expression above the median had an OS of 6.79 ± 1.68 months, substantially shorter than those with low expression (26.12 ± 3.03 months; *p* = 0.018). Univariate Cox regression analysis further supported this finding, showing that PD‐L1⁺ exosome expression was associated with a hazard ratio (HR) of 16.895 (*p* = 0.008) and 8.861 (p = 0.048) in non‐categorical and binary models, respectively (Figure S11, Supporting Information). In contrast, tissue TPS failed to predict OS, with no significant difference between patients with high TPS (≥ 50%; *n* = 8) and low TPS (≤1%; *n* = 7) (18.84 ± 4.00 months vs 18.91 ± 5.08 months; p = 0.955), and no statistical significance in Cox regression analysis (Figures S11 and S12, Supporting Information). Collectively, these findings demonstrate that PD‐L1⁺ exosomes captured using our avidity‐based platform have strong potential as prognostic and predictive biomarkers for patient outcomes following ICI treatment, outperforming conventional tissue‐based assessments.

## 3 Discussion

The dendrimer‐mediated multivalent binding platform (Figure 1) developed in this study offers a rational solution to the sensitivity limitations encountered in exosome‐based biomarker analysis. Our findings from Figure 2 demonstrate that conjugating PD‐L1‐targeting peptides to G7 PAMAM dendrimers significantly increases binding avidity and nanoscale interaction stability compared to conventional antibodies or free peptides. This is attributed to the multivalent binding effect, which enables simultaneous interactions with multiple PD‐L1 molecules, thereby enhancing binding strength and prolonging molecular interaction. While peptides are advantageous due to their synthetic accessibility, cost‐effectiveness, and superior chemical stability, their low monovalent binding affinity often limits their clinical utility.^[^ ^17^ ^]^ By leveraging a dendrimer scaffold for hierarchical presentation of peptide ligands, our platform overcame this intrinsic limitation and achieved high‐efficiency capture of PD‐L1^+^ exosomes (Figures 3 and 4).

Specifically, G7 PAMAM dendrimers were selected as the representative avidity‐based scaffold for exosome capture since they provide a highly ordered, monodisperse architecture with a dense array of terminal functional groups,^[^ ^25^ ^]^ enabling strong multivalent interactions essential for efficient exosome capture. Compared with other nanoplatforms, such as linear or hyperbranched polymers, PAMAM dendrimers afford superior control over ligand orientation and spatial distribution, thereby enhancing both sensitivity and specificity.^[^ ^26^ ^]^ Importantly, theoretical and experimental studies have shown that well‐defined surface group densities in PAMAM dendrimers are maintained up to G7, as higher generations than G7 may encounter steric hindrance that compromises structural integrity.^[^ ^27^, ^28^ ^]^ Although lower‐generation dendrimers such as G4 or G5 can also facilitate multivalent binding, our previous work with G4 dendrimers for ACE2 receptor blockade and cancer cell isolation demonstrated functional enhancement yet inferior targeting efficacy compared to G7.^[^ ^18^, ^29^ ^]^ Accordingly, G7 was chosen as the optimal balance between structural precision and maximal ligand presentation. While the effects of dendrimer generation and physicochemical properties may vary across applications, the strategy presented here offers a generalizable framework and design principles for developing dendrimer‐based or other hyperbranched polymer scaffolds for sensitive and selective detection of exosomes.

In clinical validation using serum samples (Figure 5), our platform enabled selective enrichment of PD‐L1^+^ exosomes from patients with HCC and lung cancer, highlighting its clinical translatability. Among lung cancer patients treated with ICIs, PD‐L1^+^ exosome levels prior to treatment were significantly higher in non‐responders compared to responders, demonstrating predictive value surpassing conventional tumor PD‐L1 immunohistochemistry. The elevated PD‐L1^+^ exosome levels in non‐responders are likely reflective of tumor immune evasion. As reported by Zhang et al. and corroborated by additional findings, exosomal PD‐L1 contributes to systemic immunosuppression by inhibiting T‐cell activation, promoting T‐cell apoptosis, and suppressing immune memory.^[^ ^15^, ^16^ ^]^ These studies suggest that high levels of circulating exosomal PD‐L1 can impair antitumor immunity and may explain the poor therapeutic response to ICIs in non‐responders. Therefore, PD‐L1^+^ exosomes isolated using our avidity‐based platform hold strong potential as both prognostic and predictive biomarkers for ICI response. However, the survival analysis in this study was limited by sample size and should therefore be interpreted with caution. Power analysis using the Schoenfeld formula indicated that PD‐L1⁺ exosome levels achieved statistical power of 68% and 76% for 6‐month and 1‐year survival, respectively, approaching but not reaching the conventional threshold of 80% (See the Experimental Section for the details). Validation in a larger, independent cohort will be a primary objective of our follow‐up clinical study.

In the context of HCC, prognostic assessment remains limited by the low sensitivity and specificity of conventional surveillance tools such as ultrasonography and serum AFP.^[^ ^30^ ^]^ Although our platform showed lower diagnostic performance relative to standard serum biomarkers, likely due to low PD‐L1 tissue expression in HCC,^[^ ^22^, ^23^ ^]^ it demonstrated superior prognostic relevance. These findings suggest that PD‐L1⁺ exosome levels may serve as prognostic indicators even in patients undergoing non‐immunotherapeutic treatments, highlighting the broader applicability of our platform for disease monitoring and risk stratification beyond ICI‐based therapies.

**Figure 6:**
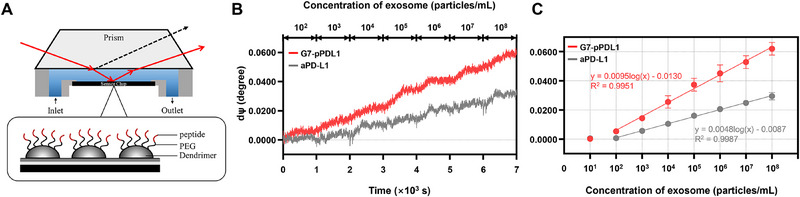
Real‐time detection of exosomes using SIS ellipsometry sensor functionalized with G7‐pPDL1. A) Schematic illustration of the SIS ellipsometry setup. Exosomes flow across a sensor chip functionalized with G7‐pPDL1 conjugates for real‐time detection based on differential changes in the ellipsometric angle Ψ (dΨ). B) Detection of exosomes derived from MDA‐MB‐231 cells at concentrations ranging from 10^1^ to 10⁸ particles mL^−1^ using the SIS ellipsometry setup. The dΨ signal is plotted over time for sensors functionalized with either G7‐pPDL1 (red) or aPD‐L1 (gray). C) Dose‐response curves showing the relationship between exosome concentration and ΔΨ. The G7‐pPDL1–functionalized SIS sensor exhibited a ≈2‐fold steeper dose‐response slope compared to aPD‐L1 between the exosome concentration of 10^2^–10^8^ particles mL^−1^ (0.0095 vs 0.0048) with a lower detection limit (9.6 × 10^1^ vs 3.7 × 10^2^ particles mL^−1^). Data represent mean ± SD.

While our study demonstrates enhanced exosome capture using a multivalent binding approach, the quantification methods employed—fluorescence intensity, BCA protein assay, and NTA—each have inherent limitations in sensitivity and specificity. To overcome these constraints and further illustrate the versatility of our platform, we integrated the G7‐pPDL1 conjugates into a solution‐immersed silicon (SIS) ellipsometry sensor for label‐free, real‐time detection (**Figure** **6**A).^[^ ^31^, ^32^, ^33^ ^]^ Exosomes derived from MDA‐MB‐231 cells were serially introduced at concentrations ranging from 10^1^ to 10^8^ vesicles mL^−1^, and the differential change in the ellipsometric angle Ψ (dΨ) was measured as a function of concentration (Figure 6B).

The G7‐pPDL1–functionalized sensor exhibited a steeper logarithmic response (slope: 0.0095) compared to the aPD‐L1–functionalized sensor (slope: 0.0048) between 10^2^ and 10^8^ vesicles mL^−1^, corresponding to a 2.04‐fold enhancement in resolution. Notably, at low exosome concentrations (from 10^1^ to 10^2^ vesicles mL^−1^), the G7‐pPDL1 surface yielded a dΨ increase of 0.00483, whereas the aPD‐L1 surface showed only a minimal change of 0.00045. As a result, when the limit of detection (LOD) was calculated as a concentration of exosomes corresponding to 3 times the standard deviation plus the baseline Ψ signal, the G7‐pPDL1 system achieved a LOD of ≈9.6 × 10^1^ vesicles mL^−1^, which was significantly lower than the aPD‐L1‐based system (≈3.7 × 10^2^ vesicles mL^−1^). Note that our system also demonstrated a good linear dynamic response with an R^2^ value of 0.995 at given exosome concentrations (Figure 6C). These results further validate the potential of our dendrimer‐peptide conjugate strategy as a universal capture platform adaptable to a range of biosensing modalities for the sensitive and specific detection of PD‐L1‐expressing exosomes.

## 4 Conclusion

In summary, we developed an avidity‐based capture platform that enables high‐affinity and selective detection of PD‐L1‐expressing exosomes, addressing key limitations of conventional exosome detection strategies, such as insufficient sensitivity and limited ability to distinguish tumor‐derived vesicles. By conjugating pPDL1 peptides onto G7 PAMAM dendrimers, the platform leverages multivalent binding interactions to enhance molecular avidity. This enhanced binding translated into improved capture of both PD‐L1‐expressing cells and exosomes, as demonstrated through molecular force spectroscopy, in vitro assays, and clinical sample analysis. Importantly, PD‐L1⁺ exosomes captured using our system effectively predicted ICI responses in lung cancer patients and exhibited superior prognostic value compared to conventional tissue‐based PD‐L1 scoring. Moreover, integration with an SIS ellipsometry sensor demonstrated the adaptability of our system for real‐time, label‐free exosome detection across a broad dynamic range. These findings highlight the potential of our avidity‐based capture strategy as a sensitive and clinically applicable exosome analysis platform for advancing precision cancer immunotherapy.

## 5 Experimental Section

### Statistical Analysis

Statistical analyses were performed using GraphPad Prism (version 10.5.0; GraphPad Software, San Diego, CA) or IBM SPSS Statistics (version 31; IBM Corp., Armonk, NY). Continuous variables—including contact angles, PD‐L1⁺ cell retention efficiencies, PD‐L1⁺ exosome capture efficiencies, exosome size, zeta potential, and PD‐L1 expression in cell line‐derived exosomes—were compared using two‐sided Student's *t*‐test (n ≥ 3 for all experiments). For AFM force mapping, data distribution was assessed with the Kolmogorov–Smirnov test, which indicated non‐normality; thus, comparisons were performed using the two‐sided Mann–Whitney *U* test.

For clinical sample analyses, tissue PD‐L1 tumor proportion score (TPS) and PD‐L1⁺ exosome enrichment efficiency was compared between responders and non‐responders using Student's *t*‐test. Receiver operating characteristic (ROC) curves were generated to evaluate the discriminatory capacity of PD‐L1⁺ exosome enrichment efficiency for distinguishing hepatocellular carcinoma (HCC) patients from healthy donors, as well as responders from non‐responders in lung cancer patients. Kaplan–Meier (KM) survival analyses and Cox regression modeling were used to estimate overall survival (OS), with survival distributions compared using the log‐rank test.

All results are reported as mean ± standard deviation (SD) unless otherwise noted. Statistical significance was defined as ^#^ *p* < 0.10, ^*^ *p* < 0.05, ^**^ *p* < 0.01, ^***^ *p* < 0.001, and ^****^ *p* < 0.0001.

Other detailed experimental procedures are provided in the Supporting Information.

## Conflict of Interest

The authors declare no conflict of interest.

## Supporting information



Supporting Information

## Data Availability

The data that support the findings of this study are available from the corresponding author upon reasonable request.
