# Supplementary material for: Avidity‐Based Capture of PD‐L1‐Expressing Exosomes via Dendrimer–Peptide Conjugates: A Nanoengineered Platform for Enhanced Prediction of Immunotherapy Response
Source: Adv Sci (Weinh). 2025 Aug 21;12(42):e09270. doi: 10.1002/advs.202509270 (PMC12622428; doi:10.1002/advs.202509270)
Supplement: Supplementary file 1 — Supporting Information [file ADVS-12-e09270-s001.pdf]

Supporting Information

Avidity-Based Capture of PD-L1-Expressing Exosomes via Dendrimer–Peptide Conjugates: A Nanoengineered Platform for Enhanced Prediction of Immunotherapy Response

*Jiah Lee, Dongjun Shin, Chae Yeon Son, Hanbit Kang, Jung Hyun Choi, Hyun Sung Park, Seha Bang, Lucia Kim, Tae Hee Lee, Hyuk Soo Eun, Michael J Poellmann, Woo-jin Jeong**, *Dong Hyung Kim**, *Jun Hyeok Lim**, *Seungpyo Hong**, *Jiyoon Bu**

Table S1. Clinical characteristics of 30 patients with hepatocellular carcinoma (HCC) and 7 healthy controls involeved in this study.

Sample ID	Age	Gender	TNM Stage	Treatment	AFP (ng/mL)	AST (IU/L)	ALT (IU/L)	Albumin (g/dL)
CNUH01	39	M	III	TACE	71.69	64	46	3.4
CNUH02	78	F	IV	Chemotherapy(Sorafenib)	12020	26	14	3.9
CNUH03	59	M	IV	Chemotherapy(Sorafenib)	5.43	32	12	2.7
CNUH04	68	F	IV	Surgery	57.75	15	10	4.1
CNUH05	65	M	I	RFA	2.88	23	29	4.1
CNUH06	81	M	II	Chemotherapy(Sorafenib)	105.6	38	12	3.5
CNUH07	64	M	I	TACE	6.02	28	19	3.4
CNUH08	71	M	II	RFA	151.07	71	15	2.5
CNUH09	68	F	II	RFA	150.6	70	29	3
CNUH10	43	M	I	RFA	29.03	47	9	3.4
CNUH11	66	M	II	RFA	72.72	33	10	4.5
CNUH12	65	M	II	RFA	11.61	28	10	3.5
CNUH13	70	F	II	RFA	6.84	35	18	3.7
CNUH14	92	F	I	RFA	1.59	17	6	3.3
CNUH15	62	F	I	RFA	11.51	85	53	3.3
CNUH16	76	M	II	RFA	4.83	37	21	3.7
CNUH17	73	F	I	RFA	10.95	27	25	4.3
CNUH18	61	F	I	RFA	3.52	21	16	3.9
CNUH19	59	F	I	RFA	1.83	27	13	3.9
CNUH20	70	M	III	RFA	8.92	31	23	3.9
CNUH21	55	M	I	RFA	2.46	53	51	4.3
CNUH22	68	M	I	RFA	26.1	38	27	3.8
CNUH23	65	M	II	RFA	201.1	36	24	4.1
CNUH24	63	M	I	TACE	14.04	25	26	4
CNUH25	64	M	II	RFA	6.11	37	24	3.6
CNUH26	62	M	I	RFA	6.32	21	21	4.3
CNUH27	58	F	I	RFA	4.1	24	9	3
CNUH28	61	M	II	Surgery	2.08	34	33	3.7
CNUH29	68	M	II	RFA	2.61	17	11	4.4
CNUH30	40	M	II	Surgery	31.16	39	55	4.1

HD01	59	F	26	21	4.5
HD02	38	M	23	18	4.9
HD03	49	F	20	23	4.8
HD04	55	F	19	10	4.7
HD05	55	F	20	18	4.2
HD06	67	F	21	18	4.8
HD07	52	F	21	23	4.2
HD08	54	F	22	33	4.6
HD09	50	M	16	13	4.6
HD10	61	M	25	34	4.7

TACE: transarterial chemoembolization; RFA: radiofrequency ablation; AFP: alpha-fetoprotein; AST: aspartate aminotransferase; ALT: alanine aminotransferase

Table S2. Clinical characteristics of 15 lung cancer patients invovled in this study.

Sample ID	Age	Gender	Histology	EGFR mutation	ICI Treated	TNM Classification				Metastasis tissue	IO Response	Best Response
						Stage	T	N	M			
INHA001	70	M	Adenocarcinoma	Negative	Pembrolizumab	IV	T4	N1	M1c	Brain, Soft tissue	R	PR
INHA002	63	M	NSCLC	Negative	Pembrolizumab	IV	T4	N3	M1c	Adrenal gland, Peritoneum, Soft tissue	R	PR
INHA003	68	M	Adenocarcinoma	Negative	Pembrolizumab	IV	T4	N3	M1a	Contralateral lung	R	PR
INHA004	48	M	Adenocarcinoma	Negative	Atezolizumab	IV	T1a	N3	M1c	Neck lymph node	R	PR
INHA005	58	F	Adenocarcinoma	Negative	Pembrolizumab	IV	Tx	N3	M1c	Extrathoracic lymph node	R	PR
INHA006	64	M	NSCLC	Negative	Pembrolizumab	IV	T4	N2	M1c	Liver, Abdominal lymph node	NR	PD
INHA007	68	M	NSCLC	Negative	Pembrolizumab	IV	T1c	N2	M1c	Brain, Contralateral lung	NR	PD
INHA008	75	M	Adenocarcinoma	Negative	Pembrolizumab	IV	T4	N2	M1c	Bone, Pleura	NR	PD
INHA009	82	F	Squamous cell carcinoma	Negative	Pembrolizumab	IV	T3	N3	M1c	Bone, Pericardium	NR	PD
INHA010	81	M	Squamous cell carcinoma	Negative	Pembrolizumab	IV	T4	N2	M1a	Pleura	NR	PD
INHA011	62	M	Squamous cell carcinoma	Negative	Pembrolizumab	IV	T4	N2	M1a	Contralateral lung	R	PR
INHA012	76	F	Adenocarcinoma	19 del	Atezolizumab	IV	T2b	N3	M1c	Brain	NR	SD
INHA013	78	F	Adenocarcinoma	Negative	Pembrolizumab	IV	Tx	N2	M1c	Brain, Abdominal lymph node	R	PR
INHA014	49	M	Adenocarcinoma	Negative	Pembrolizumab	IV	T1b	N3	M1c	Neck lymph node, Axillary lymph node	R	PR
INHA015	57	M	NSCLC	Negative	Pembrolizumab	IV	T4	N3	M1c	Lung, Brain, Liver, Adrenal	NR	PD

ICI: immune checkpoint inhibitor; R: responder; NR: non-responder; PR: partial response; SD: stable diseaes; PD: progressive disease

Table S3. Summary of OS stratified by serum biomarker levels (AFP, AST, and albumin) and PD-L1$^+$ exosomes in HCC patients. Median values were used as thresholds to define high and low expression groups.

**Biomarker**	**Overall Survival (Month)**		**Significance (*p*-value)**
	**≥ Median**	**< Median**	
PD-L1$^+$ Exosomes	65.52 ± 15.65	93.88 ± 15.32	0.0519
Serum AFP	71.41 ± 16.29	80.72 ± 12.22	0.1718
Serum AST	85.13 ± 16.86	72.04 ± 12.02	0.9514
Serum Albumin	110.06 ± 17.48	54.19 ± 11.50	0.0647

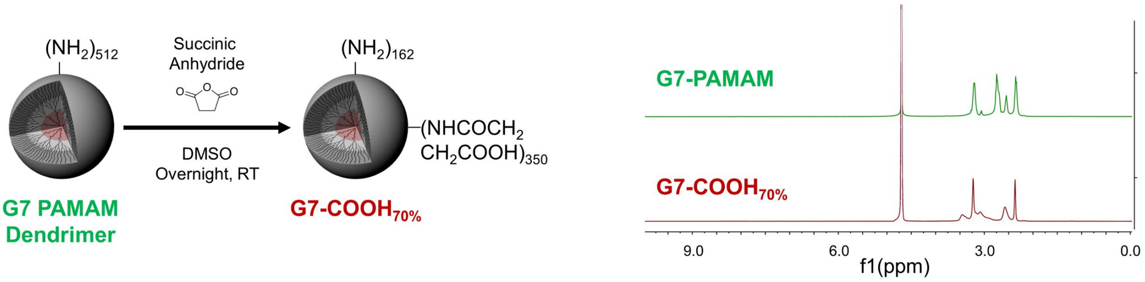

Figure S1. ^{1}H NMR spectra of G7 PAMAM dendrimer and partially carboxylated G7-$COOH_{70\%}$.

pPDL1: Ac-HGVGHGESPSGGTGTKAA (18 mer)

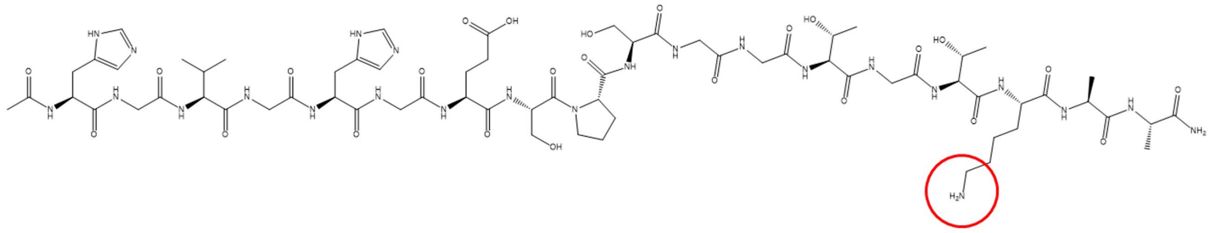

pPDL1$_{Scr}$: Ac-SEGGGHSVHTAGGAGKPT (18 mer)

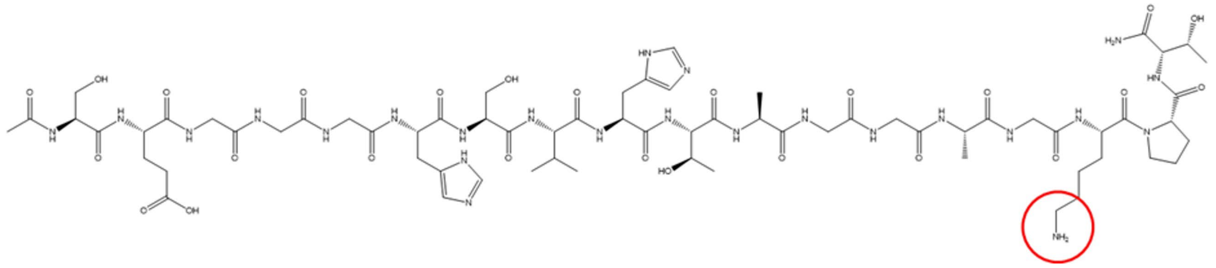

Figure S2. Chemical structures of the peptides used in this study(pPDL1) and its scrambled control(pPDL1$_{Scr}$). The side chain of Lys(K) for pPDL1 and pPDL1$_{Scr}$ marked with red circles are utilized for conjugation.

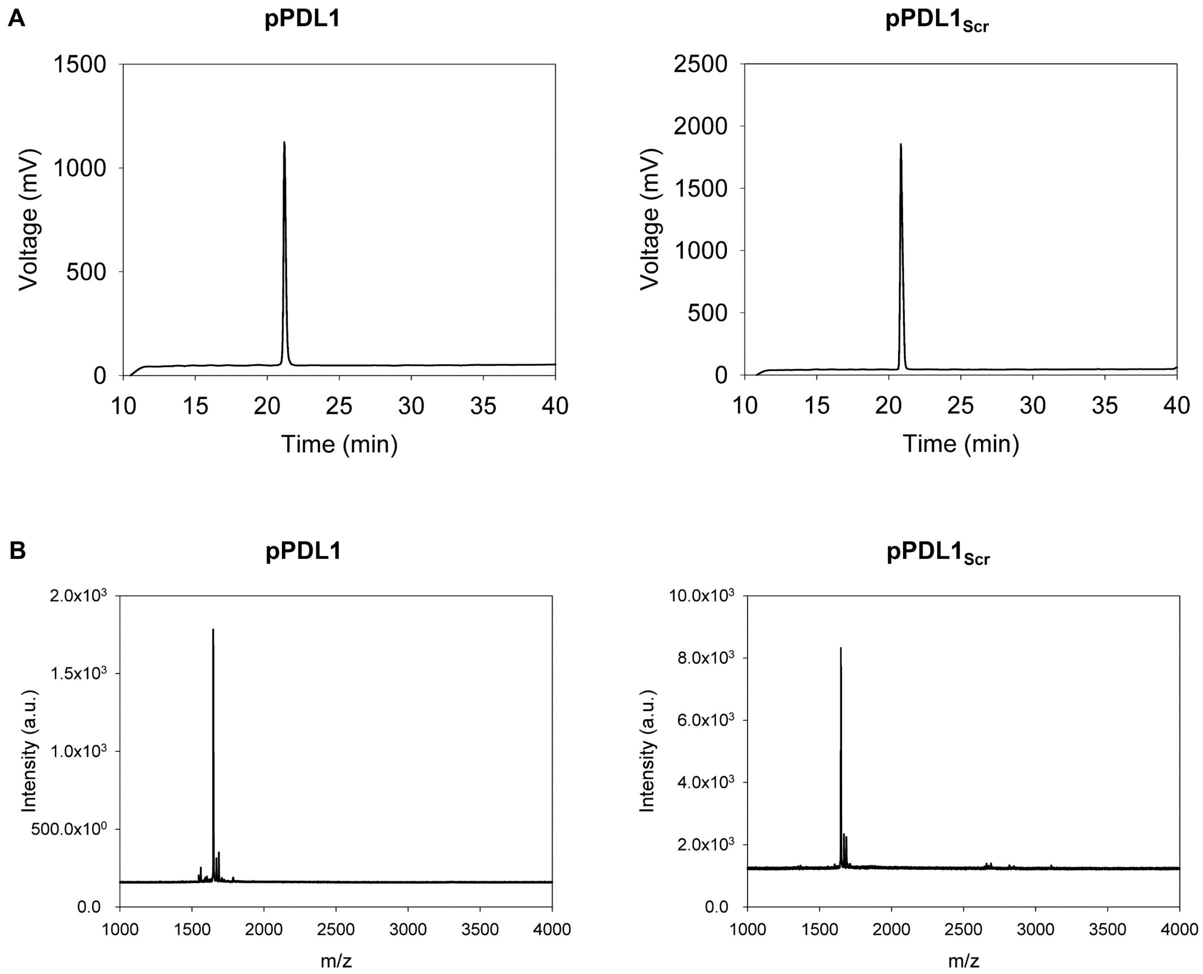

Figure S3. Characterization of synthesized peptides with HPLC and MALDI-TOF A) RP-HPLC chromatograms of purified peptides. B) MALDI-TOF spectra of pPDL1 and pPDL1$_{Scr}$ peptides.

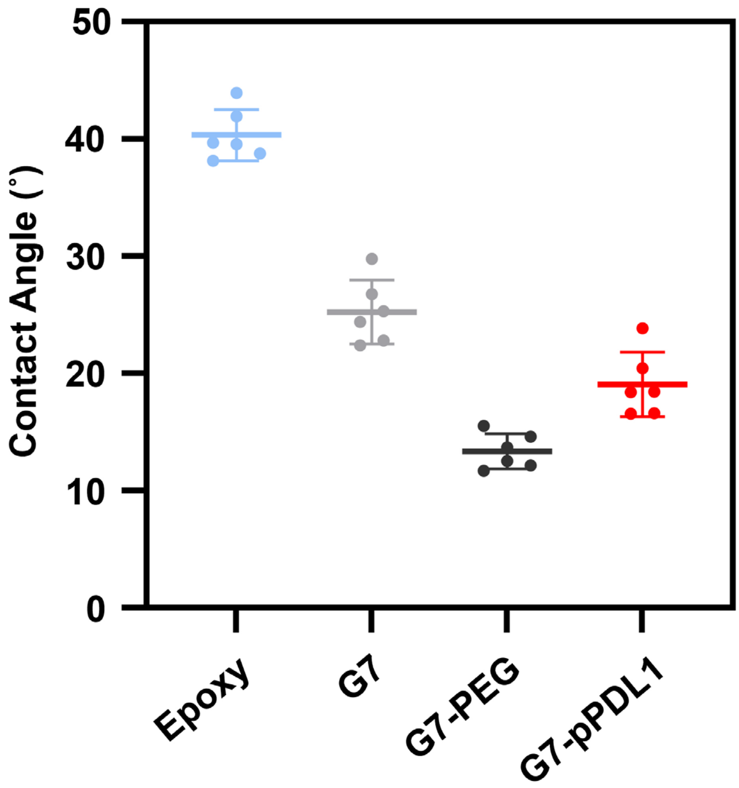

Figure S4. Contact angle measurements of epoxy-coated slides upon stepwise surface modification. Introduction of G7 and PEG progressively increased surface hydrophilicity, as indicated by decreasing water contact angles. In contrast, conjugation of the pPDL1 peptide slightly increased the contact angle, suggesting incorporation of relatively hydrophobic residues on the surface.

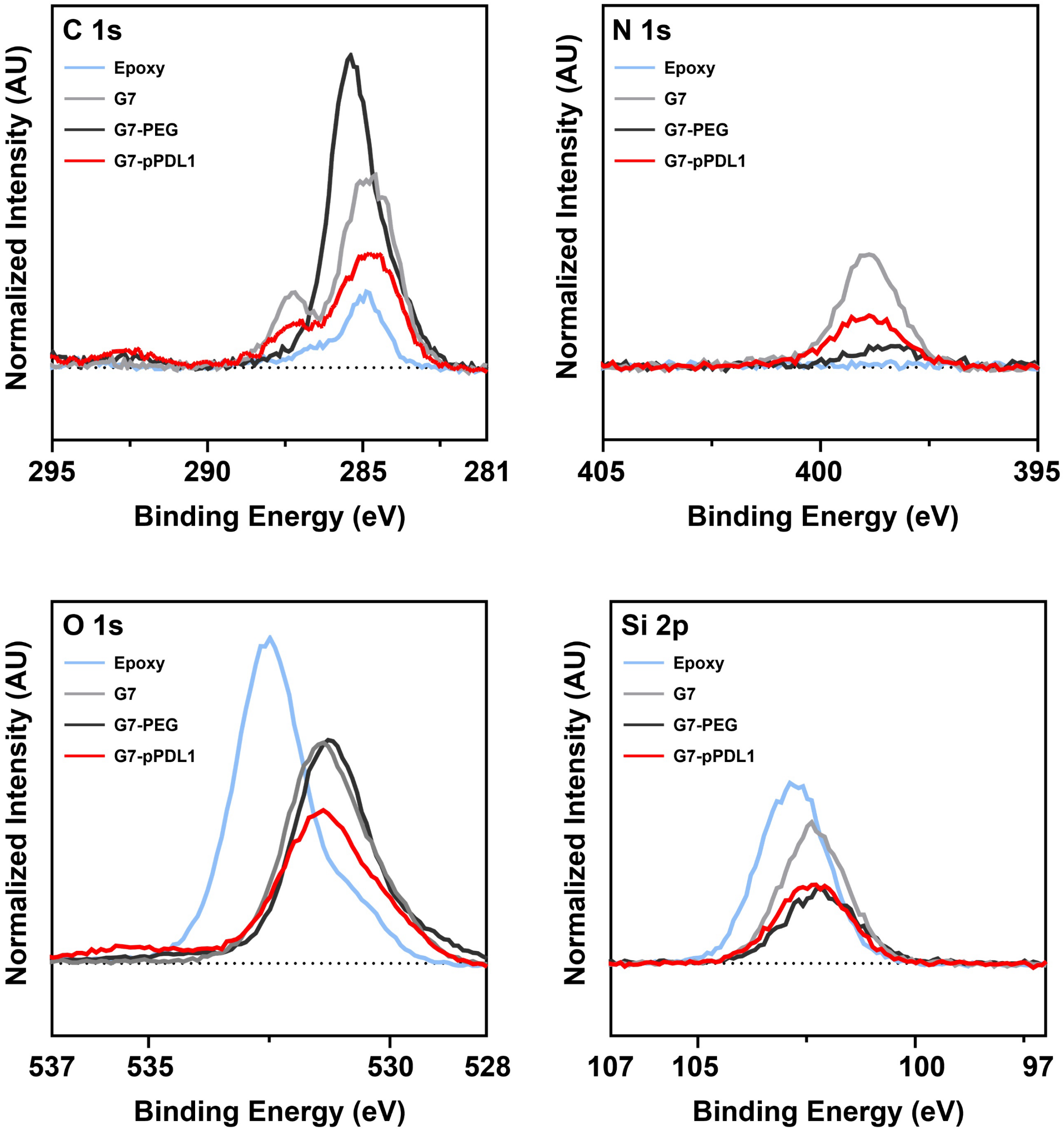

Figure S5. XPS spectra of C 1s, N 1s, O 1s, and Si 2p regions obtained from epoxy-coated glass slides following stepwise surface modification. Changes in elemental composition reflect successful conjugation of functional groups at each modification step.

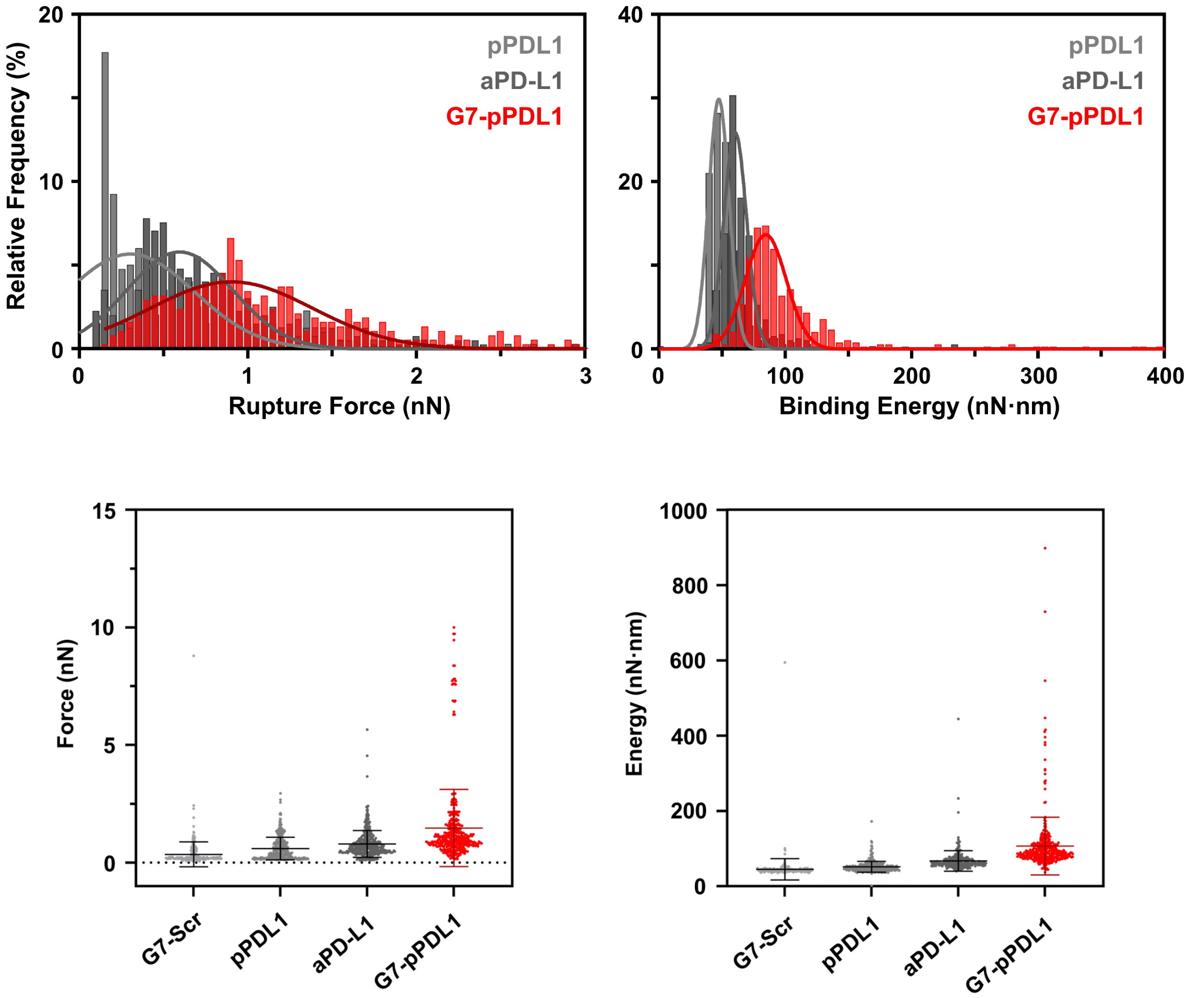

Figure S6. AFM force spectroscopy measurements showed binding force and energy between PD-L1-functionalized AFM probe and various nano receptor surfaces. G7-pPDL1 demonstrated significantly enhanced adhesion force and energy compared to pPDL1 or aPD-L1.

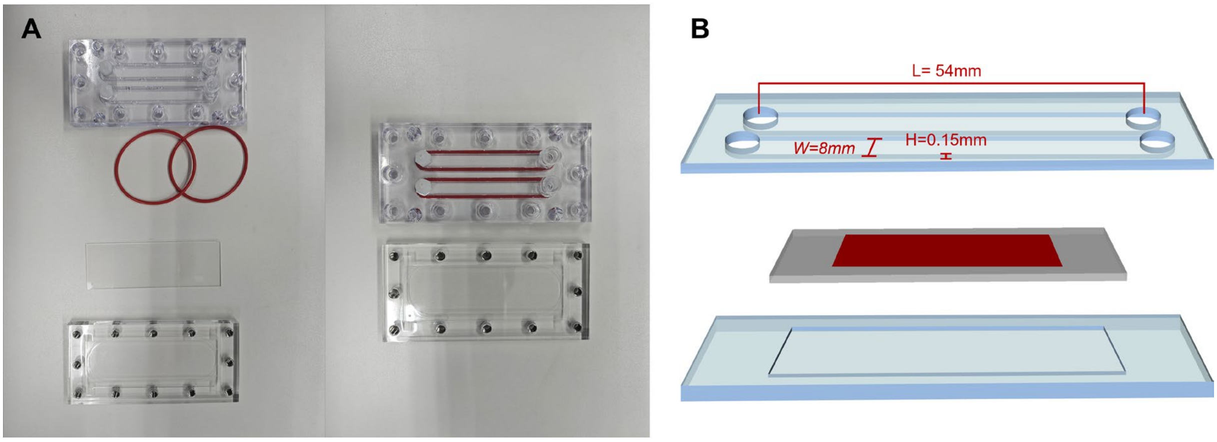

Figure S7. (**A**) Image of the custom-designed flow chamber used for the cell retention assay. (**B**) The chamber was designed with a flow path dimension of 54 mm (length) x 8 mm (width) x 0.15 mm (height)

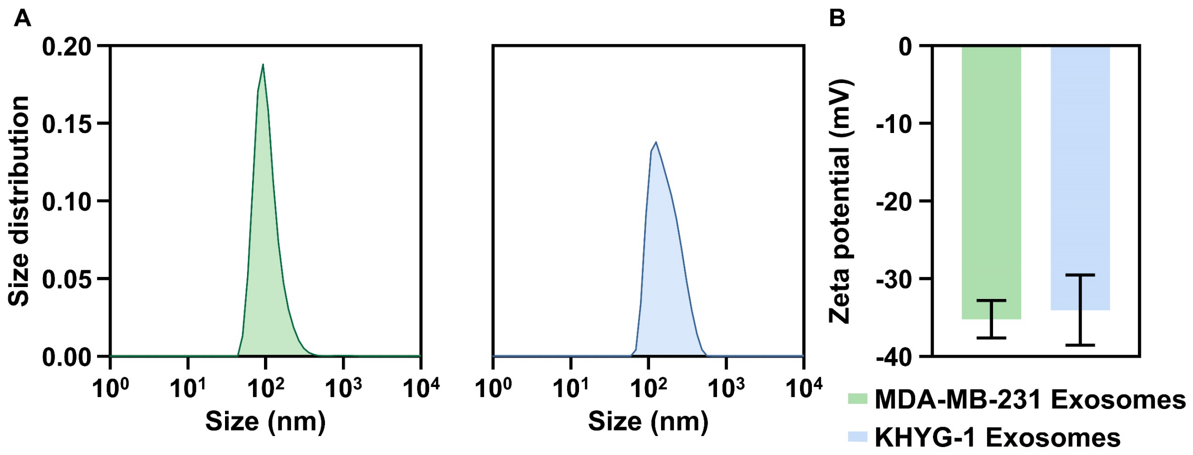

Figure S8. DLS analysis showing (**A**) Size distribution and (**B**) Zeta potential of exosomes derived from PD-L1^{High} MDA-MB-231 and PD-L1^{Negative} KHYG-1 cells.

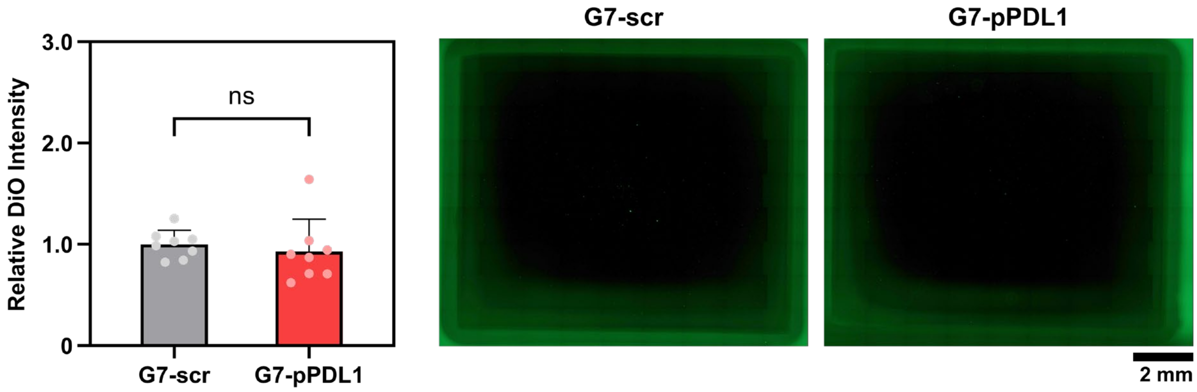

Figure S9. Evaluation of non-specific binding using DiO-labeled KHYG-1–derived exosomes spiked into human serum, following incubation on G7-pPDL1 and G7-pPDL1$_{scr}$ surfaces.

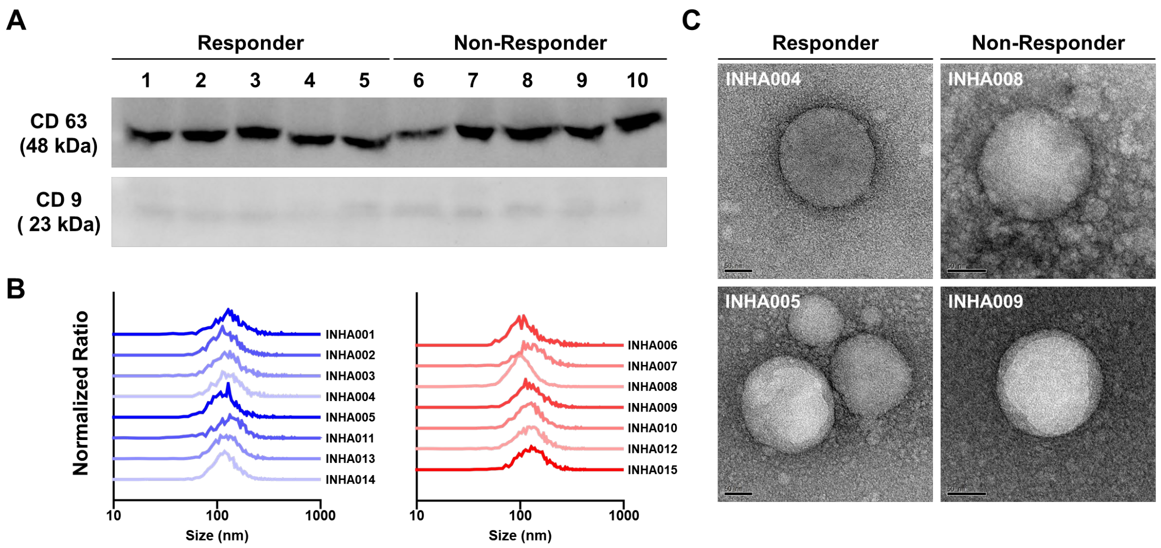

Figure S10. Characterization of exosomes isolated using ExoQuick™ prior to clinical application. (**A**) Western blot analysis confirming the presence of exosomal markers CD63 and CD9 from serum-derived exosomes. (**B**) NTA profiles showing the size distribution of isolated exosomes across multiple patient samples, consistent with expected vesicle dimensions. (**C**) TEM images of exosomes isolated from patient plasma samples (INHA004, INHA005, INHA008, and INHA009), demonstrating characteristic spherical morphology. Scale bars represent 50 nm for each image.

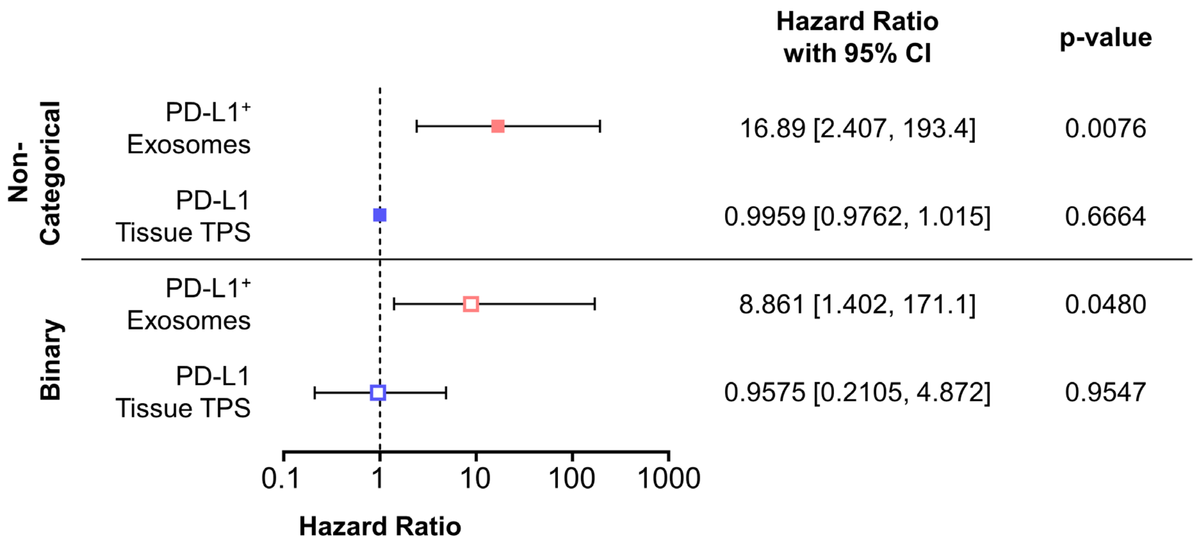

Figure S11. Forest plot analysis of hazard ratios for OS in lung cancer patients treated with ICIs. Univariate Cox regression analysis demonstrating the prognostic impact of PD-L1$^+$ exosome expression compared with tissue PD-L1 TPS. PD-L1$^+$ exosome expression was significantly associated with poorer overall survival, whereas TPS failed to show a significant prognostic correlation.

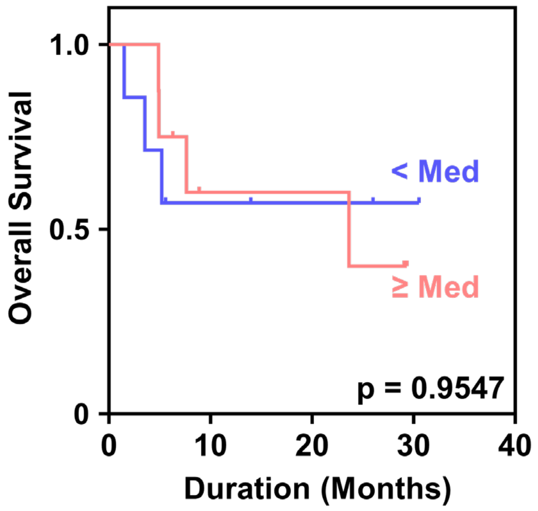

Figure S12. KM analysis of OS stratified by tissue PD-L1 TPS. Lung cancer patients treated with ICIs were grouped according to TPS (≥ 50% vs. ≤ 1%). No significant difference in OS was observed between the two groups (18.84 ± 4.00 months vs. 18.91 ± 5.08 months; p = 0.955), indicating limited prognostic value of tissue TPS.

Experimental Section

Peptide synthesis and characteriaztion: Peptides were synthesized on Rink amide MBHA resin LL (Novabiochem®, Laufelfingen, Switzerland) using standard Fmoc-based solid-phase peptide synthesis (SPPS) (**Figures S2–S4**). Automated synthesis was performed using a heat-assisted PurePep Chorus synthesizer (Gyros Protein Technologies AB, Uppsala, Sweden). Fmoc-protected L-amino acids (Novabiochem®) were coupled using 1-hydroxybenzotriazole hydrate (HOBt; Sigma-Aldrich, St. Louis, MO), O-(1H-6-chlorobenzotriazol-1-yl)-1,1,3,3-tetramethyluronium hexafluorophosphate (HCTU; Novabiochem®), and N,N-diisopropylethylamine (DIPEA; Sigma-Aldrich). N-terminal acetylation was performed using acetic anhydride (Sigma-Aldrich) and DIPEA in N,N-dimethylformamide (DMF; Thermo Fisher Scientific, Waltham, MA). Following chain assembly, peptides were cleaved from the resin and deprotected using a cocktail of 95% trifluoroacetic acid (TFA), 2.5% ethanedithiol (EDT), and 2.5% thioanisole. Cleaved peptides were precipitated by centrifugation (4,000 rpm, 10 min) in tert-butyl methyl ether (TBME) and dissolved in 10% acetonitrile. Peptides were purified by reverse-phase high-performance liquid chromatography (RP-HPLC) using a C18 column and a 0–50% linear gradient of acetonitrile in water containing 0.1% TFA at a flow rate of 2 mL/min (**Figure S3**). Molecular weights were confirmed by matrix-assisted laser desorption/ionization time-of-flight (MALDI-TOF) mass spectrometry (Bruker, Billerica, MA) using α-cyano-4-hydroxycinnamic acid (CHCA) as the matrix (**Figure S4**). The extinction coefficient at 214 nm was calculated as 29,118 $M^{-1}cm^{-1}$, and final peptide concentrations were determined by ultraviolet-visible (UV-Vis) spectrophotometry.

Dendrimer Carboxylation: Seventy percent carboxylated generation 7 (G7) polyamidoamine (PAMAM) dendrimers (Dendritech Inc., Midland, MI) were synthesized by reacting amine-terminated dendrimers with succinic anhydride, as previously described.[21] Briefly, a 10 kDa centrifugal filter unit was pre-washed twice with ddH_2O (4,000 × g, 1 min). G7 PAMAM dendrimers (50 µL) were diluted in 15 mL ddH_2O and centrifuged at 4,000 × g for 15 min. The filtrate was discarded, and the retained dendrimer solution was washed four additional times with fresh ddH_2O (15 mL each, 4,000 × g, 15 min) to remove unreacted reagents. The remaining solution was transferred to a 1.5 mL microtube, frozen at –80 °C for 2 hours, and lyophilized for 24 hours. The lyophilized dendrimers were redissolved in DMSO containing 5 mg/mL succinic anhydride (Sigma-Aldrich), and the carboxylation reaction was carried out overnight under stirring. The reaction mixture was diluted in 15 mL ddH_2O and centrifuged five times (4,000 × g, 15 min) to remove DMSO. The final product was collected, frozen (–80 °C, 2 hours),

and lyophilized for 24 hours. Partially modified G7 PAMAM dendrimers were characterized with ^1H NMR.

Surface Preparation: Schematic illustrations of the surface modification procedure are provided in **Figure 1**. Epoxy-functionalized microscope slide glass (Tekdon Incorporated, Myakka City, FL) was inserted into either four-well trays (75 mm × 25 mm) or eight-well chambers. For G7-pPDL1 immobilization, 70% carboxylated G7 PAMAM dendrimers were applied to each well at a concentration of 0.1 mg/mL in ddH_2O and incubated for over 5 hours. After washing the slides three times with ddH_2O, the carboxyl groups of the immobilized dendrimers were activated for 30 minutes using 4 μg/mL 1-ethyl-3-(3-dimethylaminopropyl)carbodiimide hydrochloride (EDC; Thermo Fisher Scientific) and 6 μg/mL N-hydroxysuccinimide (NHS; Thermo Fisher Scientific) in ddH_2O. The surfaces were then washed three times with 1× PBS (Gibco) and incubated for over 5 hours with an excess amount of NH_2-PEG_{5000}-COOH linker (1 mg/mL in 1× PBS; Biopharma PEG, Watertown, MA). Following PEG conjugation, the surfaces were wahsed once again, and the carboxyl groups of the PEG linkers were activated with 4 μg/mL EDC and 6 μg/mL NHS in ddH_2O for 30 minutes. After a final wash with 1× PBS, surfaces were incubated for more than 5 hours with pPDL1 peptide (100 nmol/mL in 1× PBS). Similarly, G7-$pPDL1_{scr}$ surface was prerpared by functionalizing $pPDL1_{scr}$ in equimolar concnetration, replacing pPDL1 during the final conjugation step. For direct peptide or antibody immobilization, PEG linkers (1 mg/mL in ddH_2O) were first conjugated to the epoxy-functionalized slides and activated using EDC and NHS as described above. The slides were then incubated for over 5 hours with either pPDL1 peptide (100 nmol/mL in 1× PBS) or anti–PD-L1 antibody (10 μg/mL in 1× PBS; CAT# SIM0009, Bio×cell, San Antonio, TX).

Contact Angle Measurement: Contact angle measurements were performed to evaluate surface wettability following each step of surface functionalization. A Phoenix 300 Touch contact angle goniometer (SEO, Suwon, Korea) was used for all measurements. Epoxy-functionalized glass slides served as the control surface, and modified surfaces were prepared by sequential immobilization of partially carboxylated G7 PAMAM dendrimers, PEG, and pPDL1 on epoxy-coated substrates. A 20 μL droplet of ddH_2O was placed on each surface using the sessile drop method, and the static contact angle was measured. Droplet images were captured during measurement and analyzed using ImageJ software.

X-ray Photoelectron Spectroscopy (XPS) Analysis: XPS was performed to analyze the surface chemical composition and functional groups present on epoxy-functionalized, G7, G7–PEG, and G7–pPDL1-modified glass slides. Measurements were carried out using a NEXSA XPS system (Thermo Scientific) equipped with a monochromatic Al Kα X-ray source (1486.6 eV). A spot size of 400 μm was used, with a pass energy of 200 eV for wide-scan spectra to assess elemental composition, and 50 eV for high-resolution scans targeting specific chemical states. Core-level spectra of the C 1s, N 1s, O 1s, and Si 2p regions were collected and analyzed to track surface modifications, including changes associated with epoxy groups, amine-functionalized dendrimers, PEG linkers, and peptide immobilization.

Surface Roughness Measurement using AFM: Surface roughness of the pPDL1- and G7-pPDL1–functionalized glass surfaces was characterized using an MFP-3D Infinity Bio-AFM system (Oxford Instruments, Santa Barbara, CA), following protocols described in our previous studies. Imaging was performed in tapping mode using a silicon probe (Bruker SNL) with a nominal spring constant of ~60 N/m. Topographical scans were analyzed to evaluate changes in surface morphology resulting from peptide and dendrimer conjugation.

AFM Force Spectroscopy: AFM-based force spectroscopy was employed to assess the nanoscale binding interactions between PD-L1–functionalized probes and various capture surfaces. To mimic exosome binding, AFM probes (PNP-TR-Au-20; NanoWorld, Neuchâtel, Switzerland) were functionalized with recombinant human PD-L1 protein (Cat. No. 156-B7; R&D Systems, Minneapolis, MN). Probes were initially treated with a mixture of 1.9 mg/mL 5 kDa methoxy-PEG-thiol and 0.1 mg/mL 7.5 kDa carboxyl-PEG-thiol (Jenkem, Plano, TX) in ddH_2O. After rinsing with ddH_2O, the carboxyl groups were activated using 5.7 mg/mL EDC and 6.6 mg/mL NHS. The activated probes were then incubated for 1 hour at 4 °C with recombinant PD-L1 at a concentration of 5 μg/mL to complete conjugation. The spring constants of the probes were calibrated using the thermal noise method. Force–distance (FD) mapping was performed over a 2.0 × 2.0 μm^2 area using a 20 × 20 array. FD curves were acquired on G7-pPDL1, G7-pPDL1$_{scr}$, pPDL1, and aPD-L1–functionalized surfaces using PD-L1–modified probes. Each force cycle involved a 2 μm approach and retraction at a speed of 2 μm/s. Discrete unbinding events were identified as abrupt changes in the retraction force profile exceeding 3,000 pN. From each FD curve, the maximum adhesion force and adhesion energy were extracted and spatially mapped onto 20 × 20 pixel grids for comparative analysis across the surfaces.

Cell Preparation: Two human breast cancer cell lines (MDA-MB-231 and MCF-7) and one human natural killer/T-cell lymphoma line (KHYG-1) were used in this study. MDA-MB-231 cells and MCF-7cells were kindly provided by Capio Biosciences Korea, while KHYG-1 cells were provided by H.J. Kim at Inha University. MDA-MB-231 and MCF-7 cells were cultured in Dulbecco's Modified Eagle Medium (DMEM; Gibco), supplemented with 10% (v/v) fetal bovine serum (FBS; Gibco) and 1% (v/v) penicillin–streptomycin (P/S; Gibco). KHYG-1 cells were maintained in RPMI medium (Gibco) supplemented with 10% FBS and 1% P/S. All cells were incubated at 37 °C in a humidified atmosphere containing 5% CO_2. MDA-MB-231 and MCF-7 cells were cultured as adherent monolayers, while KHYG-1 cells were maintained in suspension. Cells were expanded in T-75 flasks for in vitro retention assays and in T-175 flasks for exosome collection until reaching 60–80% confluence. All experiments were performed using cells at passages below 15. For fluorescent labeling, cells were washed three times with 1× PBS (Gibco) and detached using 0.25% trypsin-EDTA (Gibco), followed by centrifugation at 1,500 rpm for 5 min. KHYG-1 cells were collected directly from the culture medium without enzymatic detachment. All cells were incubated in 1 mL of complete medium containing 4 μL of calcein AM (Invitrogen) for 30 minutes at 37 °C. Following incubation, cells were washed three times by centrifugation (1,500 rpm, 5 min) and resuspended in fresh complete medium. Final cell suspensions were adjusted to a concentration of 5×10^5 cells/mL for downstream applications.

In Vitro Flow Chamber Cell Retention Assay: Functionalized glass slides were mounted in flow chambers for retention analysis. Fluorescently labeled cells were introduced into the chamber using a syringe pump (New Era Pump Systems Inc., Farmingdale, NY) at a flow rate of 500 μL/min. Following a 10-minute incubation at room temperature to allow cell attachment, fresh complete medium was flowed through the chamber at 100 μL/min to remove unbound cells. Retention efficiency was defined as the ratio of cells remaining on the surface after washing to the total number of cells initially introduced. To evaluate PD-L1 binding selectivity, the retention of MDA-MB-231 (PD-L1^{High}), MCF-7 (PD-L1^{Low}), and KHYG-1 (PD-L1^{Negative}) cells were compared across G7-pPDL1 surface.

Immunofluorescent Analysis of PD-L1 Expression: PD-L1 expression on MDA-MB-231, MCF-7, and KHYG-1 cells was analyzed using Alexa Fluor® 555-conjugated anti–PD-L1 antibody (Abcam, Cambridge, UK). Adherent MDA-MB-231 and MCF-7 cells were gently washed three

times with 1× PBS (Gibco), detached using 0.25% trypsin-EDTA (Gibco), and centrifuged at 1,500 rpm for 5 minutes. KHYG-1 suspension cells were collected directly from the culture medium without enzymatic treatment. Collected cells were incubated for 1 hour in 200 μL of 1× PBS containing Alexa Fluor® 555 anti–PD-L1 antibody at a 1:100 dilution (2 μL antibody). The cell concentration was adjusted to 5 × 10^4 cells/mL during staining. After incubation, cells were centrifuged at 1,500 rpm for 5 minutes and resuspended in 200 μL of 1× PBS to remove excess unbound antibody. Labeled cells were transferred to a 96-well plate and allowed to settle for 30 minutes. Fluorescent images were acquired using a fluorescence microscope under appropriate excitation/emission settings (555 nm/565 nm). ImageJ software was used to quantify the fluorescence intensity of PD-L1 expression.

Exosome Collection from Cell Lines: After cells reached 60–80% confluency in T-175 flasks, the culture medium was replaced with exosome-depleted medium containing 5 wt.% exosome-depleted FBS (Gibco). After 48 hours of incubation, the conditioned medium was collected and subjected to two rounds of centrifugation at 2,000 rpm for 10 minutes to remove cells and large cellular debris. The supernatant was subsequently filtered through a 0.22 μm vacuum filter and ultracentrifuged at 28,700 rpm using a SW 55Ti rotor (Beckman Coulter, Brea, CA). Following ultracentrifugation, the supernatant was carefully removed, and the resulting exosome pellet was resuspended in 1× PBS. Exosome concentration was determined by NTA, following the following the manufacturer's instructions.

Exosome Capture and Analysis: Exosomes derived from cultured cells or patient plasma samples were used for surface capture experiments. Plasma-derived exosomes were diluted 1:10 in 1× PBS, and 100 μL of the diluted solution was added to each well of an eight-well chamber slide. The slides were pre-coated with G7-pPDL1, pPDL1, aPD-L1, or G7-pPDL1$_{scr}$ surfaces for comparative exosome capture. Samples were incubated at 37 °C for 3 hours to allow exosome binding to the functionalized surfaces. Following incubation, each well was washed three times with 250 μL of 1× PBS to remove unbound exosomes. The amount of exosomes captured on each surface was quantified using three independent methods: NTA, Micro BCA protein assay, and DiO fluorescence analysis. Each assay was performed according to its standard protocol to assess the relative capture efficiency of the various surface modifications.

NTA: The particle concentration and size distribution of uncaptured exosomes were analyzed using a ZetaView® Nanoparticle Tracking Analyzer (PMX230; Particle Metrix, Meerbusch,

Germany) operating in scatter mode, following the manufacturer's guidelines. Each sample was recorded under standardized acquisition parameters. Particle size (mean diameter, nm) and concentration (particles/mL) were calculated using ZetaView software. All measurements were performed in triplicate to ensure reproducibility and minimize technical variability. Note that serum-derived exosomes isolated using ExoQuick™ were also characterized by NTA under the same protocol to confirm particle size distribution and concentration.
BCA assay: The amount of protein associated with captured exosomes was quantified using the Micro BCA Protein Assay Kit (Thermo Scientific). A BSA standard curve ranging from 0 to 200 μg/mL was prepared by serial dilution of a 2 mg/mL BSA stock solution. The working reagent (WR) was freshly prepared by mixing Reagents A, B, and C at a 25:24:1 ratio. For each sample or standard, 100 μL of WR was added to 100 μL of solution in a 96-well microplate. Plates were incubated at 37 °C and subsequently cooled to room temperature. Absorbance was measured at 562 nm using a microplate reader, and protein concentrations were interpolated from the standard curve. All measurements were performed in duplicate.
DiO fluorescence imaging: Captured exosomes were labeled using Vybrant™ DiO Cell-Labeling Solution (Invitrogen). The DiO working solution was prepared by diluting 5 μL of DiO stock into 1 mL of PBS. Each well received 100 μL of the dye solution and was incubated at 37 °C for 30 minutes. After staining, wells were washed three times with 250 μL of 1× PBS to remove unbound dye. Fluorescent imaging was performed using an inverted fluorescence microscope at 5× magnification. Exposure settings were kept constant for all samples, and brightness/contrast adjustments were uniformly applied across images. Image analysis was performed to evaluate fluorescent signal intensity as a proxy for exosome capture.

Patient Recruitment: Human serum samples were collected from 15 lung cancer patients at Inha University Hospital prior to the initiation of immune checkpoint inhibitor (ICI) therapy, under institutional review board (IRB) approval (IRB No. 2005-03-001). Tumor response was evaluated using RECIST v1.1 criteria. Based on the best response, patients were categorized as responders (PR) or non-responders (SD or PD). OS was defined as the time from the initiation of ICI therapy to death from any cause. The PD-L1 TPS was determined by immunohistochemistry using validated anti-PD-L1 antibodies (SP263 [Roche Tissue Diagnostics, Oro Valley, AZ]).[S1] In addition, serum samples from 30 HCC patients were obtained from Chungnam National University Hospital (CNUH-2020-08-049-001 and CNUH2020-10-088-023) from National University Hospital biobank of Chungnam and

Chungbuk. Written informed consent was obtained from all participants prior to sample collection.

Clinical Sample Processing: Exosomes were isolated from plasma using the ExoQuick™ Plasma Prep and Exosome Precipitation Kit (System Biosciences, Palo Alto, CA) according to the manufacturer's protocol. Briefly, 250 µL of plasma was mixed with 63 µL of ExoQuick™ solution, vortexed, and incubated at 4 °C for at least 30 minutes to induce exosome precipitation. Samples were then centrifuged at 1,500 × g for 30 minutes, and the resulting exosome pellet was resuspended in 1× PBS. All procedures were performed on ice or at 4 °C to ensure sample integrity. Resuspended exosomes were diluted to a final volume of 200 µL and applied to each well of an eight-well chamber at a concentration of approximately 12.8 µg/mm² for 3 hours. PD-L1$^+$ exosome enrichment efficiency was calculated by comparing the amount of total exosomal protein captured on G7-pPDL1 surfaces to that captured on negative control surfaces (G7-pPDL1$_{scr}$).

Transmission Electron Microscopy Analysis: Transmission electron microscopy (CM200; PHILIPS, Amsterdam, Netherlands) was employed to examine the morphology of exosomes derived from serum samples or cell lines. For sample preparation, 2 µL of exosome suspension was applied onto a carbon-coated copper grid (200 mesh; Electron Microscopy Sciences, Hatfield, PA, USA) and air-dried at room temperature. Negative staining was performed using 2 µL of Uranyless EM stain solution (Electron Microscopy Sciences), a non-radioactive alternative to uranyl acetate. Excess stain was carefully removed, and the grids were completely dried prior to imaging with the CM200 transmission electron microscope.

Western Blotting: Serum-derived exosomes isolated using ExoQuick™ Plasma Prep and Exosome Precipitation Kit were lysed in RIPA buffer, and protein concentrations were determined by Micro BCA Protein Assay. Equal amounts of protein were separated on SDS–polyacrylamide gels and transferred onto PVDF membranes under wet transfer conditions. Membranes were blocked with 5% skim milk for 1 h at room temperature and incubated overnight at 4 °C with primary antibodies against CD63 (Catalog No. 353039, BioLegend) and CD9 (Catalog No. ab236630, abcam). Following three washes, membranes were incubated with HRP-conjugated secondary antibodies for 1 h at room temperature. Protein bands were visualized using a a chemiluminescent reagent, SuperSignal™ West Femto Maximum

Sensitivity Substrate (Thermo Fisher Scientific) and imaged using ChemiDoc™ MP Imaging System (Bio-Rad).

Sensor Chip Fabrication for SIS Measurement: n-Type silicon wafers (11.55 mm × 11.5 mm) were cleaned by sequential rinsing with acetone and ethanol, followed by nitrogen blow-drying to eliminate surface contaminants. To hydroxylate the silicon surface, wafers were treated with piranha solution (3:1 v/v mixture of sulfuric acid and hydrogen peroxide) and subsequently functionalized by incubating in 2% (v/v) APTES (3-aminopropyltriethoxysilane) in anhydrous ethanol for 12 hours at room temperature. Wafers were thoroughly rinsed with anhydrous ethanol to remove residual reagents and then mounted onto a dual-prism assembly for DP-SIS (dual-prism solution-immersed silicon) sensor construction.

For antibody immobilization, anti–PD-L1 antibodies were covalently attached to the silane-modified surface at a concentration of 1 µg/mL using NHS ester chemistry. Unreacted active sites were blocked by injecting a 0.5% (w/v) BSA solution in PBS.

For G7-pPDL1 conjugate immobilization, 70% carboxylated G7 PAMAM dendrimers (G7-$COOH_{70}$%) were first immobilized onto the silanized surface at 1 µg/mL via NHS ester chemistry. Next, 10 nmol/mL of NH_2–PEG_{500}–COOH was conjugated using standard EDC/NHS coupling. Finally, 10 nmol/mL of pPDL1 peptides were immobilized using the same EDC/NHS chemistry. Remaining reactive sites were blocked with 0.5% (w/v) BSA in PBS. All surface modifications were performed at a constant flow rate of 30 µL/min.

SIS Measurement for PD-L1-Expressing Exosome Detection: Exosome samples were diluted in PBS containing 0.1% (v/v) SDS (SDS–PBS) to prepare concentrations ranging from 10^1 to 10^8 EVs/mL on a logarithmic scale. Samples were sequentially injected onto the receptor-immobilized sensor surface at a flow rate of 100 µL/min. Binding events between exosomes and the immobilized ligands were recorded in real time, with responses expressed in ellipsometric angle changes (ΔΨ).

Statistical Power Analysis: The estimated statistical power for survival curve comparisons was calculated using the Schoenfeld formula,[S2] which offers an analytical approach for estimating the power of the log-rank test under the proportional hazards assumption. The formula is as follows:

$$Z_\beta = \frac{\sqrt{d} \times \ln(HR)}{\sqrt{1/[p(1-p)]}} - Z_{\frac{\alpha}{2}}$$

where Z_β is the standard normal deviate corresponding to the statistical power ($1-\beta$), d is the total number of observed events (deaths) at the specified time point, HR is the hazard ratio of the PD-L1$^+$ exosome enrichment rate estimated by univariate Cox regression analysis, and $Z_{\alpha/2}$ is the critical value for a two-sided significance level α (1.96 for α = 0.05). The parameter p denotes the proportion of patients classified into the PD-L1$^+$ exosome^{High} group (> median) at the specified time point. Patients who were censored before the specified timepoint were excluded from the analysis for that timepoint.

Statistical power was then determined as $\Phi(Z_\beta)$, where Φ denotes the cumulative distribution function (CDF) of the standard normal distribution. Power calculations were performed for both 6-month and 1-year survival endpoints, comparing the PD-L1$^+$ exosome^{High} and PD-L1$^+$ exosome^{Low} groups.

Note that in the 6-month survival analysis, one patient censored prior to 6 months was excluded. Similarly, for the 1-year survival analysis, three patients censored prior to 12 months were excluded.

References for Supporting Information

[S1] E. A. Eisenhauer, P. Therasse, J. Bogaerts, L. H. Schwartz, D. Sargent, R. Ford, J. Dancey, S. Arbuck, S. Gwyther, M. Mooney, L. Rubinstein, L. Shankar, L. Dodd, R. Kaplan, D. Lacombe, J. Verweij, European Journal of Cancer 2009, 45, 228-247.

[S2] D. A. Schoenfeld, Biometrika 1981, 68, 316-319.
